# Comparison of antifungal drugs in the treatment of invasive pulmonary aspergillosis: a systematic review and network meta-analysis

**DOI:** 10.3389/fmicb.2024.1504826

**Published:** 2024-12-02

**Authors:** Jing Cheng, Hedong Han, Wenwen Kang, Zijin Cai, Ping Zhan, Tangfeng Lv

**Affiliations:** ^1^Department of Respiratory and Critical Care Medicine, Nanjing Jinling Hospital, Affiliated Hospital of Medical School, Nanjing University, Nanjing, China; ^2^Department of Respiratory and Critical Care Medicine, Jinling Hospital, Nanjing Medical University, Nanjing, China

**Keywords:** invasive pulmonary aspergillosis, voriconazole, isavuconazole, posaconazole, amphotericin B

## Abstract

**Background:**

Voriconazole, isavuconazole, and amphotericin (AmB) formulations are currently recommended to treat invasive pulmonary aspergillosis (IPA). We aimed to estimate the efficacy of different antifungal drugs in the initial treatment of IPA.

**Methods:**

We included all available randomized controlled trials (RCTs) evaluating first-line treatments for IPA by searching PubMed, Medline, EMBASE, the Cochrane Library, and the ClinicalTrials.gov database. We performed a network meta-analysis to compare the relative efficacy of different drugs in treating IPA. The primary outcomes were the overall response and all-cause mortality (ACM).

**Results:**

Eight studies were identified that compared different drugs including voriconazole, isavuconazole, posaconazole, anidulafungin, liposomal AmB (L-AmB) at standard, high and low doses (3-5 mg/kg/d; 10 mg/kg/d; 1 mg/kg/d), AmB deoxycholate (dAmB) and amphotericin B colloidal dispersion (ABCD). We found that second-generation triazole antifungal drugs containing voriconazole, isavuconazole, and posaconazole exhibited significantly superior overall response to dAmB and ABCD. Voriconazole was ranked as the best drug on network rank analysis. We found no difference in efficacy between triazole antifungals and L-AmB. A combination of voriconazole with anidulafungin, isavuconazole and voriconazole showed significantly better safety than dAmB.

**Conclusion:**

The efficacy of second-generation triazole antifungal drugs for the first-line treatment of IPA is comparable with L-AmB and is better than both dAmB and ABCD. Isavuconazole may show better safety than voriconazole and posaconazole. Combination therapy with voriconazole and anidulafungin may serve as an alternative option for IPA patients with limited drug tolerance.

**Systematic review registration:**

https://inplasy.com/.

## 1 Introduction

Aspergillus is a saprophytic mold that commonly causes fungal infections in various areas of the body, particularly the lungs, resulting in invasive pulmonary aspergillosis (IPA) (Hajjeh and Warnock, 2001). IPA has been found to impact immunocompromised patients, leading to increased rates of morbidity and mortality. In addition, it has recently been recognized as a severe complication of influenza or coronavirus disease 2019 (COVID-19) in individuals who appear to have a normally functioning immune system (Lamoth and Calandra, 2022). The primary treatment options for IPA include azoles, lipid formulations of amphotericin B, amphotericin B, and echinocandins (Ledoux and Herbrecht, 2023).

In the past, dAmB was proven to be effective in treating aspergillosis (Stevens et al., 2000). Since the 1990s, lipid formulations of amphotericin B, such as L-AmB and ABCD, have been considered to be more selective because of less nephrotoxicity compared to dAmB (Tiphine et al., 1999; Hamill, 2013). Now, the guidelines demonstrate that second-generation triazole antifungal drugs are preferred as the standard of care for treating invasive aspergillosis (IA) (Patterson et al., 2016; Tissot et al., 2017; Ullmann et al., 2018). Pulmonary infection is the most common site of infection in IA. European Confederation of Medical Mycology and the European Respiratory Society recommended voriconazole or isavuconazole as the first-line treatment of IPA and L-AmB (3-5 mg/kg) as an alternative for salvage therapy (Ullmann et al., 2018). However, the treatment for IPA still needs to be improved due to the presence of adverse events, drug–drug interactions, and antifungal resistance (Neofytos et al., 2010; Andes et al., 2016). For example, the use of voriconazole in IPA may be associated with hepatotoxic disease, neurological and visual disturbances, phototoxic reactions skin disease (Benitez and Carver, 2019). In recent years, some RCTs have compared the effectiveness of voriconazole with isavuconazole and voriconazole with posaconazole for treating IPA. These trials provided substantial evidence endorsing posaconazole and isavuconazole as initial treatment options for IPA patients (Maertens et al., 2016; Maertens et al., 2021). However, no RCTs have reported the difference in efficacy between isavuconazole and posaconazole. Additionally, several studies suggest that combination therapy for IPA may yield potential benefits for certain patients (Singh et al., 2006; Caillot et al., 2007; Marr et al., 2015). However, the comparison of efficacy between combination therapy and monotherapy remains unclear.

A network meta-analysis of isavuconazole trials has indicated that the effectiveness of isavuconazole is similar to both L-AmB (3-5 mg/kg or 10 mg/kg) and voriconazole, and it outperforms dAmB (Herbrecht et al., 2018). Another meta-analysis suggested that combining liposomal amphotericin B with caspofungin could be a viable alternative for treating invasive aspergillosis (IA) and recommended second-generation triazole antifungal drugs as the primary therapy (Liu et al., 2024). However, there is insufficient evidence for using combination drugs as first-line treatment for anti-pulmonary aspergillosis, and there is a lack of comparative data on adverse reactions among different triazole drugs. To facilitate informed treatment decisions for patients with IPA, we undertake a comprehensive network meta-analysis. The patient population of our study is immunocompromised patients with proven or probable IPA. The interventions include antifungal agents such as isavuconazole, posaconazole, amphotericin B lipid formulations, and echinocandins. We compare voriconazole, commonly used in clinical practice, with these interventions. The primary outcome is the efficacy of different antifungal drugs in IPA, including the overall response and all-cause mortality. Our analysis provides more comprehensive information on common antifungal agents in initially treating IPA may guide the selection of interventions for clinicians.

## 2 Methods

The network meta-analysis was conducted following the Preferred Reporting Items for Systematic Reviews and Meta-analyses for Network Meta-analysis (PRISMA-NMA, doi: 10.1136/bmj.n71). The protocol of the study had been registered in the International Platform of Registered Systemic Review and Meta-analysis Protocols (INPLASY) under the registration number INPLASY202380105 (doi: 10.37766/inplasy2023.8.0105).

### 2.1 Search strategy

We searched PubMed, Medline, EMBASE, and the Cochrane Central Register of Controlled Clinical Trials until 30 May 2023 to identify all relevant articles on the efficacy of IPA treatment. We employed the MESH terms “Invasive Pulmonary Aspergillosis,” “Therapeutics,” and “Clinical trials as a topic.” and their free text terms in our search strategy. The detailed search strategy is presented in Supplementary Table S8. In addition, The reference lists of the included studies and previous reviews were screened for additional articles.

### 2.2 Study inclusion criteria

**Figure 1:**
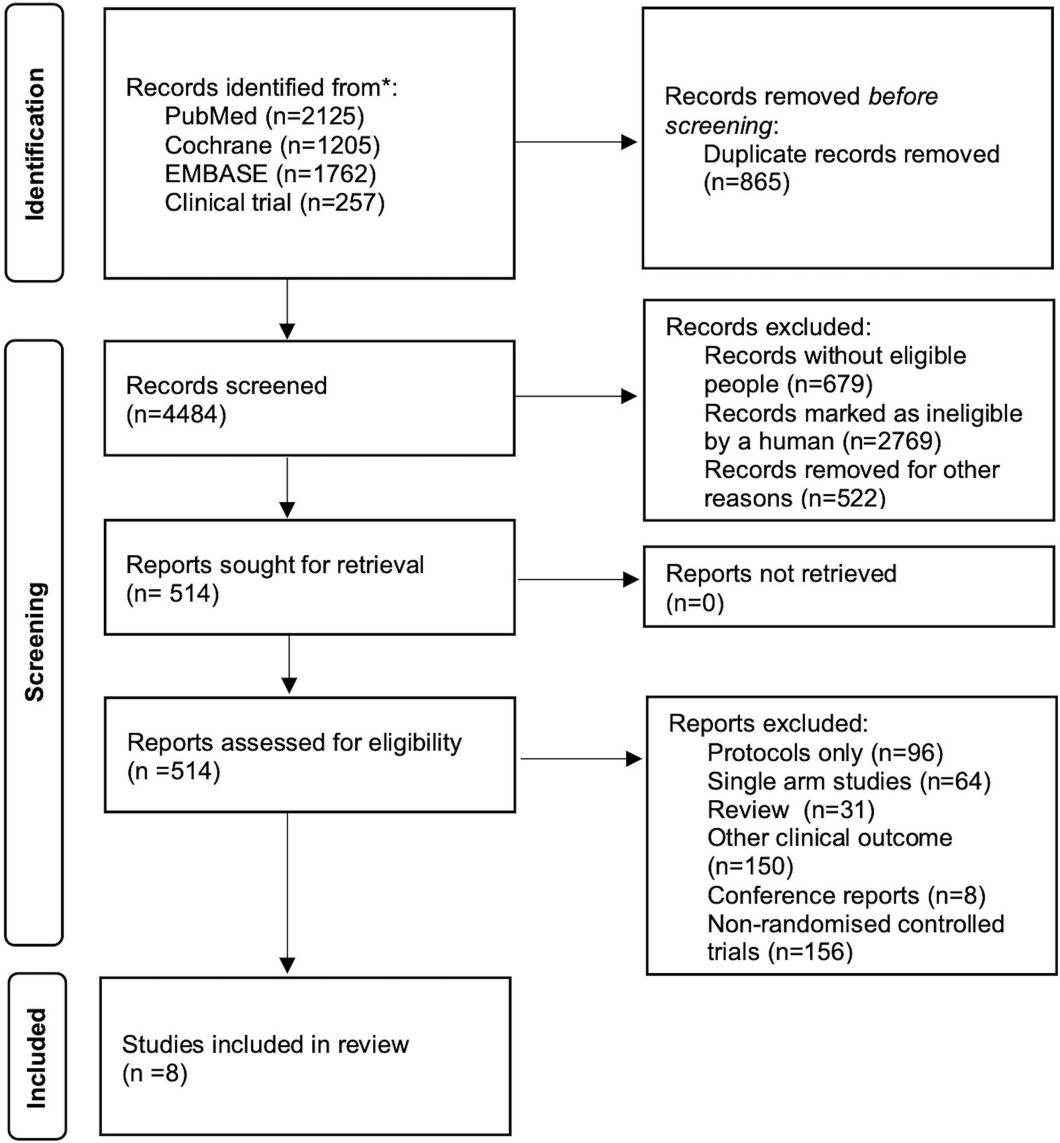
Flow diagram of study selection.

Two independent investigators (CJ and HHD) screened and identified available RCTs for inclusion (Figure 1). We included RCTs that focused on the initial treatment of patients with probable or proven IPA, specifically comparing the efficacy of different therapeutic drugs for IPA. Studies that solely consisted of case reports, studies that did not report on clinical outcomes and studies with only a single control arm were excluded from our analysis.

### 2.3 Data extraction

Two investigators independently extracted data from each relevant study as follows: (a) publication details (first author, year of publication, region); (b) study design (RCT); (c) the underlying disease of patients, total number of patients and number of participants in each arm; (d) use and dosage of drugs; (e) treatment response; and (f) adverse events or serious adverse events. During the data extraction process, any disagreements that arise are resolved through discussions.

### 2.4 Quality assessment

The risk of bias of the included studies was assessed by version 2 of the Cochrane risk-of-bias tool31 for RCTs (Supplementary Figure S1). We use the GRADE approach to rate the certainty of evidence from our included studies (Supplementary Table S9). The following domains were assessed: (1) risk of bias, (2) inconsistency, (3) indirectness, (4) imprecision, (5) publication bias, and (6) other considerations. Two researchers conducted the quality assessment independently, and in case of any disagreement, a third person was involved to resolve it.

### 2.5 Clinical outcomes

The pre-specified outcomes for the study included the overall response (complete and partial response, as defined in each study), all-cause mortality (ACM), and the rate of adverse events. The overall response was analyzed as the primary outcome. They were all reported as odds ratios (ORs) with associated 95% confidence intervals (CIs).

### 2.6 Statistical analyses

For each outcome, we initially conducted a frequentist meta-analysis to estimate treatment effects for each direct pairwise comparison. Heterogeneity was assessed by estimating variance across studies. In our analysis, we found that each comparison of treatments consisted of only one study and no heterogeneity was observed. Therefore, we chose a fixed-effect model within a frequentist framework to conduct the analysis using the “network” package in Stata statistical software version 14.2. We then used the variance method to compare the differences in treatment effects between multiple interventions and sort them according to effect size (Lu and Ades, 2004; Rouse et al., 2017).

The comparative analysis of different treatments was carried out by constructing league (Tables 1, 2). The surface under the cumulative ranking (SUCRA) method was selected to evaluate ranking probabilities and assess each treatment schedule based on their overall response rate and ACM. Each intervention was ranked based on its estimated effect, with probability values calculated for each ranking position and SUCRA values derived from cumulative ranking probabilities (Salanti et al., 2011). A higher SUCRA statistic (up to 1) indicates a greater likelihood that a specific drug will achieve the highest ranking in the network meta-analysis. We developed a ranking program that displays the probability of rank for each treatment (Supplementary Tables S1, S2; Supplementary Figures S1, S2). We also conducted an analysis of adverse events for each treatment regimen and documented specific adverse symptoms associated with them (Supplementary Tables S3, S4). We performed the sensitivity analysis by excluding the study (Ellis et al., 1998) with poor quality and conducted a publication bias assessment (Supplementary Tables S6, S7; Supplementary Figures S4, S5).

## 3 Results

### 3.1 Characteristics and quality of the included studies

**Figure 2:**
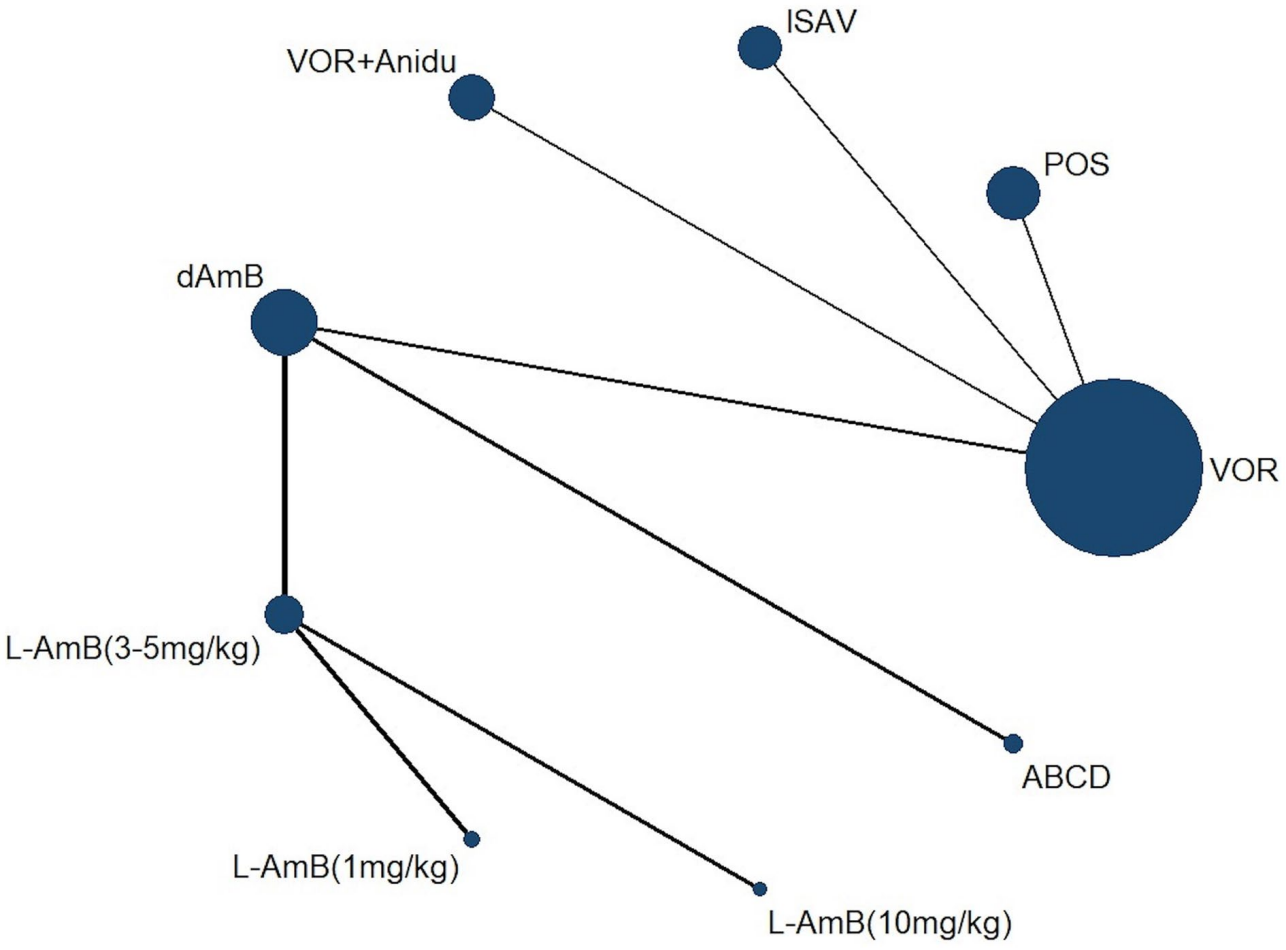
Network forest plot comparing outcomes of different antifungal drugs in patients with IPA.

A total of 4,484 records were identified and subsequently screened. Among these, 8 RCTs were included, involving a total of 1,431 patients. The 9 treatments included in the analysis were voriconazole, posaconazole, isavuconazole, combination therapy of voriconazole and anidulafungin, dAMB, L-AMB at 3-5 mg/kg/d, L-AMB at 1 mg/kg/d, L-AMB at 10 mg/kg/d, and ABCD. Figure 2 shows the network forest plot comparing outcomes of different antifungal drugs in patients with IPA. Tables 3, 4 present the essential characteristics and findings of the studies included in our analysis. We identified two studies with high certainty of evidence, five studies with moderate certainty of evidence, and one study with low certainty of evidence from Supplementary Table S9.

**Table 3:** Studies included for this analysis and characteristics of enrolled patients with invasive aspergillosis (IA).

Study	Country	Treatment	Primary underlying conditions/diseases	Number of patients included	Proportion of aspergillus	Lung as the site of infection
Maertens et al. (2021)	International	VOR	Neutropenia^†^ (48%); allogeneic HSCT (24%); use of T-cell immunosuppressants (39%); use of corticosteroids (35%)	171	all	97%
		POS	Neutropenia^†^ (46%); allogeneic HSCT (24%); use of T-cell immunosuppressants (42%); use of corticosteroids (40%);	163	all	96%
Maertens et al. (2016)	International	VOR	Neutropenia^†^ (67.8%); allogeneic BMT/HSCT (20%); use of T-cell immunosuppressants (42%); use of corticosteroids (15%); AML (49%); ALL (9%); Lymphoma (9%); AA (7%); CLL (5%); CML (3%); MDS (5%); MM (3%); COPD (1%); diabetes mellitus (0%)	129	86%^‡^	95%
		ISAV	Neutropenia^†^ (63.2%); allogeneic BMT/HSCT (21%); use of T-cell immunosuppressants (43%); use of corticosteroids (19%); AML (38%); ALL (12%); Lymphoma (13%); AA (3%); CLL (4%); CML (3%); MDS (9%); MM (2%); COPD (2%); diabetes mellitus (2%)	143	84%^‡^	89%
Marr et al. (2015)	International	VOR	Neutropenia^†^; allogeneic HSCT (29.6%); AML (30.3%); ALL (13.4%); Lymphoma (9.2%); AA (0.7%); CLL (5.6%); CML (0.7%); MDS (4.9%)	142	all	Not reported
		VOR + Anidu	Neutropenia^†^ (57.8%); allogeneic HSCT (32.6%); AML (34.8%); ALL (8.9%); Lymphoma (8.9%); AA (0.7%); CLL (3.7%); CML (0%); MDS (1.5%)	135	all	Not reported
Herbrecht et al. (2002)	International	VOR	Neutropenia^†^ (50.3%); allogeneic HSCT (22.9%); AML (35.8%); ALL (8.4%); other hematologic malignancy (11.7%); solid organ transplant (6.1%); other nonmalignant disease^§^ (7.8%)	124	all	85%
		dAmB	Neutropenia^†^ (49.4%); allogeneic HSCT (20.7%); AML (38.4%); ALL (7.3%); other hematologic malignancy (15.2%); solid organ transplant (3.7%); other nonmalignant disease ^c^ (9.8%)	113	all	88%
Leenders et al. (1998)	The Netherlands	L-AmB (5 mg/kg/d)	Neutropenia^†^ (94%); acute nonlymphocytic leukaemia/MDS (56%); ALL (19%); CL (6%); BMT (13%)	26	78%^‡^	81%
		dAmB	Neutropenia^†^ (88%); acute nonlymphocytic leukaemia/MDS (59%); ALL (9%); CL (9%); BMT (15%)	29	76%^‡^	85%
Ellis et al. (1998)	International	L-AmB (4 mg/kg/d)	Neutropenia^†^ (82%); AML (59%); ALL (22%); MM/MDS/AA/ aplastic anemia (9%); NHL (6%)	46	all	81%
		L-AmB (1 mg/kg/d)	Neutropenia^a^ (86%); AML (54%); ALL (15%); MM/MDS/AA/ aplastic anemia (24%); NHL (7%)	41	all	86%
Cornely et al. (2007)	Europe and Australia	L-AmB (3 mg/kg/d)	Neutropenia^†^ (62%); leukaemia (69%); AML (36%); lymphoma (13%); allogeneic HSCT (17.8%)	36	96%^‡^	80%
		L-AmB (10 mg/kg/d)	Neutropenia^†^ (63%); leukaemia (68%); AML (34%); lymphoma (18%); allogeneic HSCT (16%)	30	98%^‡^	79%
Bowden et al. (2002)	The United States	dAmB	bone marrow transplant (40.7%); hematologic malignancy (64%); solid tumor (12.8%); solid organ transplant (3.5%); COPD (11.6%); diabetes mellitus (8.1%)	53	all	65%
		ABCD	Bone marrow transplant (43.2%); hematologic malignancy (75%); solid tumor (4.5%); solid organ transplant (5.7%); COPD (8%); diabetes mellitus (4.5%)	50	all	67%

VOR, voriconazole; POS, Posaconazole; ISAV, isavuconazole; Anidu: anidulafungin; dAmB, deoxycholate amphotericin B; L-AmB, liposomal amphotericin B; ABCD, amphotericin B colloidal dispersion; IA, invasive aspergillosis; HSCT, haematopoietic stem-cell transplantation; BMT, bone marrow transplantation; AML, acute myeloid leukaemia; ALL, acute lymphoblastic leukaemia; CML, chronic myeloid leukaemia; CLL, chronic lymphocytic leukaemia; AA, aplastic anaemia; MDS, myelodysplastic syndrome; MM, multiple myeloma; CL, chronic leukaemia; COPD, chronic obstructive pulmonary disease. ^†^It is defined as absolute neutrophil count < 0.5 × 109/L at baseline. ^‡^Some patients infected with other non-Aspergillus molds were enrolled in the Maertens et al. (2016) study, the Leenders et al. (1998) study and the Cornely et al. (2007) study. ^§^Mostly high-dose steroid-treated or human immunodeficiency virus–positive patients.

**Table 4:** Information on treatment and clinical outcomes in identified studies.

Study	Treatment	Application and Dosage	The median duration of treatment	Number of favorable response^†^; time point	Number of all-cause deaths; time point	Adverse events
Maertens et al. (2021)	VOR	Day 1: 6 mg/kg i.v. / 300 mg p.o. BID; Day2-84: 4 mg/kg i.v. / 200 mg p.o. BID	64 days (1–81 days)	79; 84 days	53; 84 days	increased ALT, AST, or alkaline phosphatase, hallucination, increased γ-glutamyltransferase peptidase, nausea and blurred vision
	POS	Day 1: 300 mg i.v. / p.o. BID; Day 2–84: 300 mg QD	67 days (1–81 days)	69; 84 days	56; 84 days	increased AST or ALT, nausea, hypokalaemia, and vomiting
Maertens et al. (2016)	VOR	Day1: 6 mg/kg i.v. BID; Day2: 4 mg/kg i.v. BID; Day3-84: 4 mg/kg i.v. / 200 mg p.o. BID	47 days (13–83 days)	47; 84 days	48; 84 days	Gastrointestinal disorders, infections and infestation, skin and subcutaneous tissue disorders, eye disorders and hepatobiliary disorders
	ISAV	Day1-2: prodrug 372 mg i.v. TID; Day3-84200 mg i.v. / p.o. QD	45 days (13–83 days)	50; 84 days	43; 84 days	Gastrointestinal disorders and infections and infestation
Marr et al. (2015)	VOR	Day 1: 6 mg/kg i.v. BID; Day2-7: 4 mg/kg BID; Day8-42: 300 mg p.o. BID	42 days (1–48 days)	61; 42 days	55; 84 days	Eye disorders, psychiatric disorders, and skin and subcutaneous tissue disorders
	VOR + Anidu	Day1: VOR 6 mg/kg i.v. BID + Anidu 200 mg i.v.; Day2-7: VOR 4 mg/kg BID + Anidu 100 mg i.v. QD; Day8-42^‡^: 300 mg p.o. BID + Anidu 100 mg i.v. QD;	14 days (1–29 days)	44; 42 days	39; 84 days	Gastrointestinal disorders, nervous system disorders, hepatobiliary disorders
Herbrecht et al. (2015)	VOR	Day 1: 6 mg/kg i.v. BID; Day 2–8: 4 mg/kg i.v. BID; Day 9–84: 4 mg/kg i.v. / 200 mg p.o. BID	77 days (2–84 days)	62; 84 days	37; 84 days	Hepatic abnormalities, metabolism disorders, and gastrointestinal disorders
	dAmB	Day 1–84: 1–1.5 mg/kg i.v. QD	10 days (1–84 days)	29; 84 days	51; 84 days	Renal impairment, hypokalemia and fever, chills, anaphylaxis, asthenia, or myalgia
Leenders et al. (1998)	L-AmB	Day 1–14: 5 mg/kg/d i.v. QD	5.5 days (0–98 days)	18; EOT	5; EOT	Hypokalaemia, increased bilirubin, and fever or chills
	dAmB	Day 1–14: 1 mg/kg/d i.v. QD	6.5 days (0–36 days)	17; EOT	11; EOT	Nephrotoxicity, fever or chills, and hypokalaemia
Ellis et al. (1998)	L-AmB	Until EOT^§^: 4 mg/kg/d i.v. QD	19 days (3–70 days)	22; EOT	31; EOT	Renal toxicity, headache, nausea, diarrhea, rash *et al*
	L-AmB	Until EOT^§^: 1 mg/kg/d i.v. QD	18 days (2–71 days)	26; EOT	24; EOT	
Cornely et al. (2011)	L-AmB	Day 1–14: 3 mg/kg/d i.v. QD	15 days (1–60 days)	15; 84 days	17; 84 days	Significantly higher rates of nephrotoxicity and hypokalemia within the high-dose group
	L-AmB	Day 1–14: 10 mg/kg/d i.v. QD	14 days (1–57 days)	14; 84 days	15; 84 days	
Bowden et al. (2002)	dAmB	Day 1–42: 1–1.5 mg/kg/d i.v. QD	14.5 days (1–87 days)	31; EOT	24; EOT	Renal toxicity, hypoxia, and chill or fever
	ABCD	Day 1–42: 6 mg/kg/d i.v. QD	13 days (1–357 days)	24; EOT	18; EOT	Significantly lower renal toxicity and chill or fever

VOR, voriconazole; POS, Posaconazole; ISAV, isavuconazole; Anidu: anidulafungin; dAmB, deoxycholate amphotericin B; L-AmB, liposomal amphotericin B; ABCD, amphotericin B colloidal dispersion; i.v., intravenous injection; p.o., per os by mouth; QD, once daily; BID, twice daily; TID, three times daily; ALT, alanine aminotransferase; AST, aspartate aminotransferase; EOT: end of treatment. ^†^Defined as a successful response (complete or partial overall response) assessed by the data review committee. ^‡^Voriconazole was maintained for 42 days, and anidufungin was maintained for 14 to 28 days. ^§^The therapy was given for at least 14 days, or death or a toxic event necessitating withdrawal of L-AmB occurred.

### 3.2 Overall response

**Figure 3:**
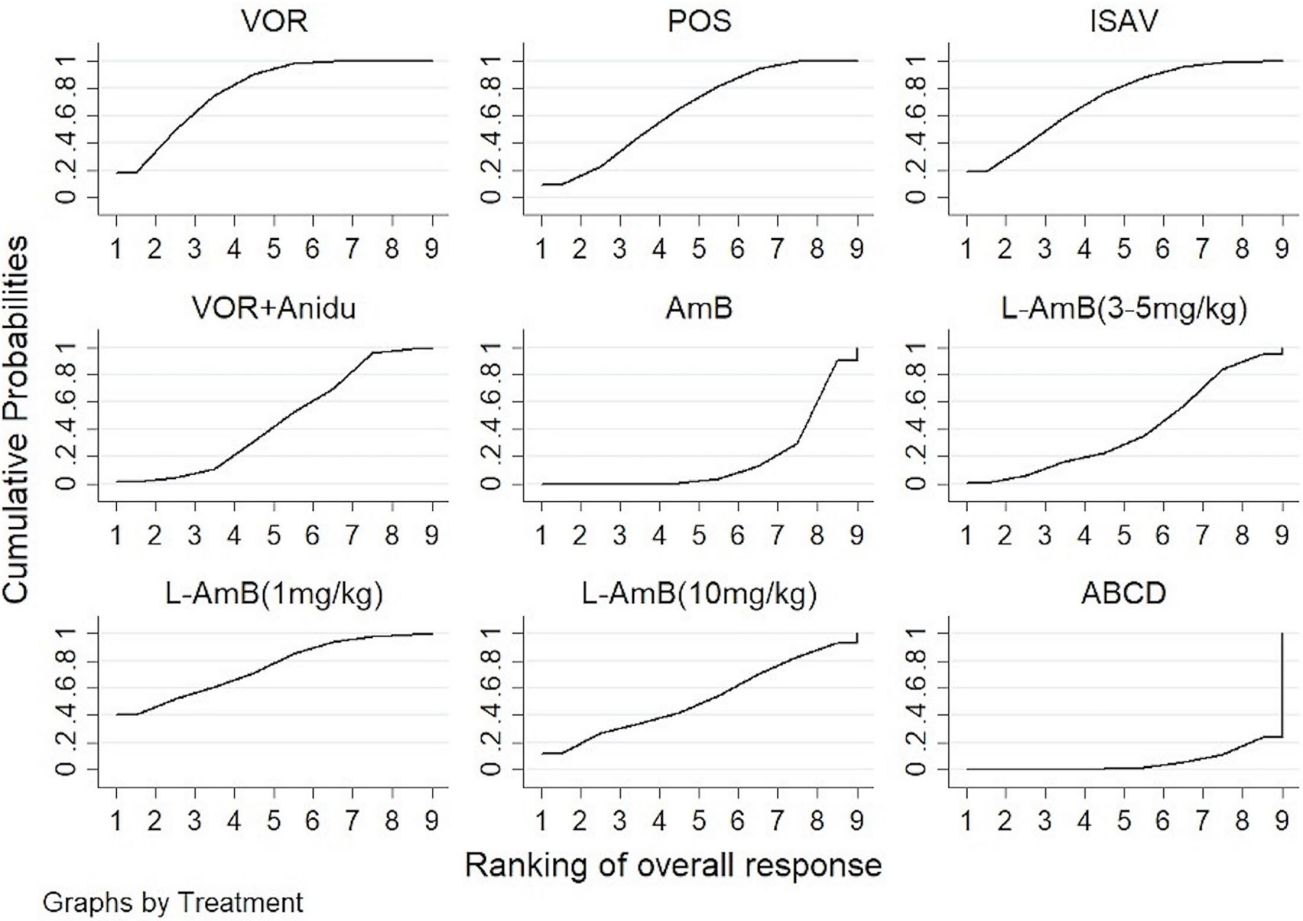
The ranking profile of comparable treatments on overall response for patients with invasive pulmonary aspergillosis (IPA). The X-axis represents ranking, and the Y-axis cumulative probabilities. (VOR, voriconazole; POS, Posaconazole; ISAV, isavuconazole; Anidu: anidulafungin; AmB, deoxycholate amphotericin B; L-AmB, liposomal amphotericin B; ABCD, amphotericin B colloidal dispersion).

Voriconazole, isavuconazole, and posaconazole all demonstrated significantly superior efficacy compared to dAMB and ABCD (Table 1). Voriconazole yielded a superior overall response against dAMB (OR 2.90; 95% CI: 1.67–5.02) and ABCD (OR 4.42; 95% CI: 1.70–11.47). Isavuconazole achieved a superior overall response against dAMB (OR 2.72; 95% CI:1.30–5.70) and ABCD (OR 4.15;95% CI:1.42–12.15). And posaconazole yielded a superior overall response against dAMB (OR 2.48; 95% CI:1.23–4.98) and ABCD (OR 3.78; 95% CI:1.33–10.76). There is no statistically significant difference between triazole antifungals and L-AmB. It also showed no difference in overall response among standard, high and low doses of L-AmB. Based on the ranking profiles, voriconazole achieved the highest ranking with a SUCRA value of 79% and a mean rank of 2.7. It was followed by L-AmB at 1 mg/kg/d with a SUCRA value of 74.9% and isavuconazole with a SUCRA value of 72.1% (Figure 3; Supplementary Table S1).

**Table 1:** Pooled odds ratios (95% confidence intervals) for the number of favorable responses/the total number of patients included.

VOR	1.04 (0.23,4.69)	0.94 (0.57,1.54)	0.85 (0.55,1.32)	0.67 (0.14,3.26)	0.64 (0.39,1.05)	0.55 (0.16,1.90)	**0.35 (0.20,0.60)**	**0.23 (0.09,0.59)**
0.96 (0.21,4.36)	L-AmB(1 mg/kg)	0.90 (0.18,4.43)	0.82 (0.17,3.96)	0.65 (0.18,2.38)	0.62 (0.13,3.03)	0.53 (0.22,1.25)	0.33 (0.08,1.36)	0.22 (0.04,1.09)
1.07 (0.65,1.75)	1.11 (0.23,5.42)	ISAV	0.91 (0.47,1.76)	0.72 (0.14,3.75)	0.68 (0.34,1.38)	0.58 (0.15,2.23)	**0.37 (0.18,0.77)**	**0.24 (0.08,0.71)**
1.17 (0.76,1.80)	1.21 (0.25,5.83)	1.10 (0.57,2.12)	POS	0.79 (0.15,4.04)	0.75 (0.39,1.44)	0.64 (0.17,2.39)	**0.40 (0.20,0.81)**	**0.26 (0.09,0.75)**
1.49 (0.31,7.22)	1.54 (0.42,5.67)	1.40 (0.27,7.31)	1.27 (0.25,6.54)	L-AmB(10 mg/kg)	0.96 (0.18,4.99)	0.82 (0.31,2.17)	0.51 (0.12,2.26)	0.34 (0.06,1.79)
1.56 (0.95,2.54)	1.62 (0.33,7.90)	1.46 (0.73,2.93)	1.33 (0.69,2.56)	1.05 (0.20,5.47)	VOR + Anidu	0.85 (0.22,3.24)	0.54 (0.26,1.12)	0.35 (0.12,1.03)
1.82 (0.53,6.31)	1.89 (0.80,4.47)	1.71 (0.45,6.51)	1.56 (0.42,5.80)	1.23 (0.46,3.25)	1.17 (0.31,4.45)	L-AmB(3-5 mg/kg)	0.63 (0.21,1.92)	0.41 (0.11,1.60)
**2.90 (1.67,5.02)**	3.00 (0.74,12.26)	**2.72 (1.30,5.70)**	**2.48 (1.23,4.98)**	1.95 (0.44,8.55)	1.86 (0.89,3.88)	1.59 (0.52,4.84)	dAmB	0.66 (0.30,1.43)
**4.42 (1.70,11.47)**	4.58 (0.92,22.88)	**4.15 (1.42,12.15)**	**3.78 (1.33,10.76)**	2.97 (0.56,15.83)	2.84 (0.97,8.29)	2.42 (0.62,9.43)	1.53 (0.70,3.33)	ABCD

Data in each cell are odd ratios (95% confidence intervals) for comparing row-defining treatment versus column-defining treatment. Odds ratios more than one favor row-defining treatment. The results in bold are significant. VOR, voriconazole; POS, Posaconazole; ISAV, isavuconazole; Anidu: anidulafungin; dAmB, deoxycholate amphotericin B; L-AmB, liposomal amphotericin B; ABCD, amphotericin B colloidal dispersion.

### 3.3 All-cause mortality

**Figure 4:**
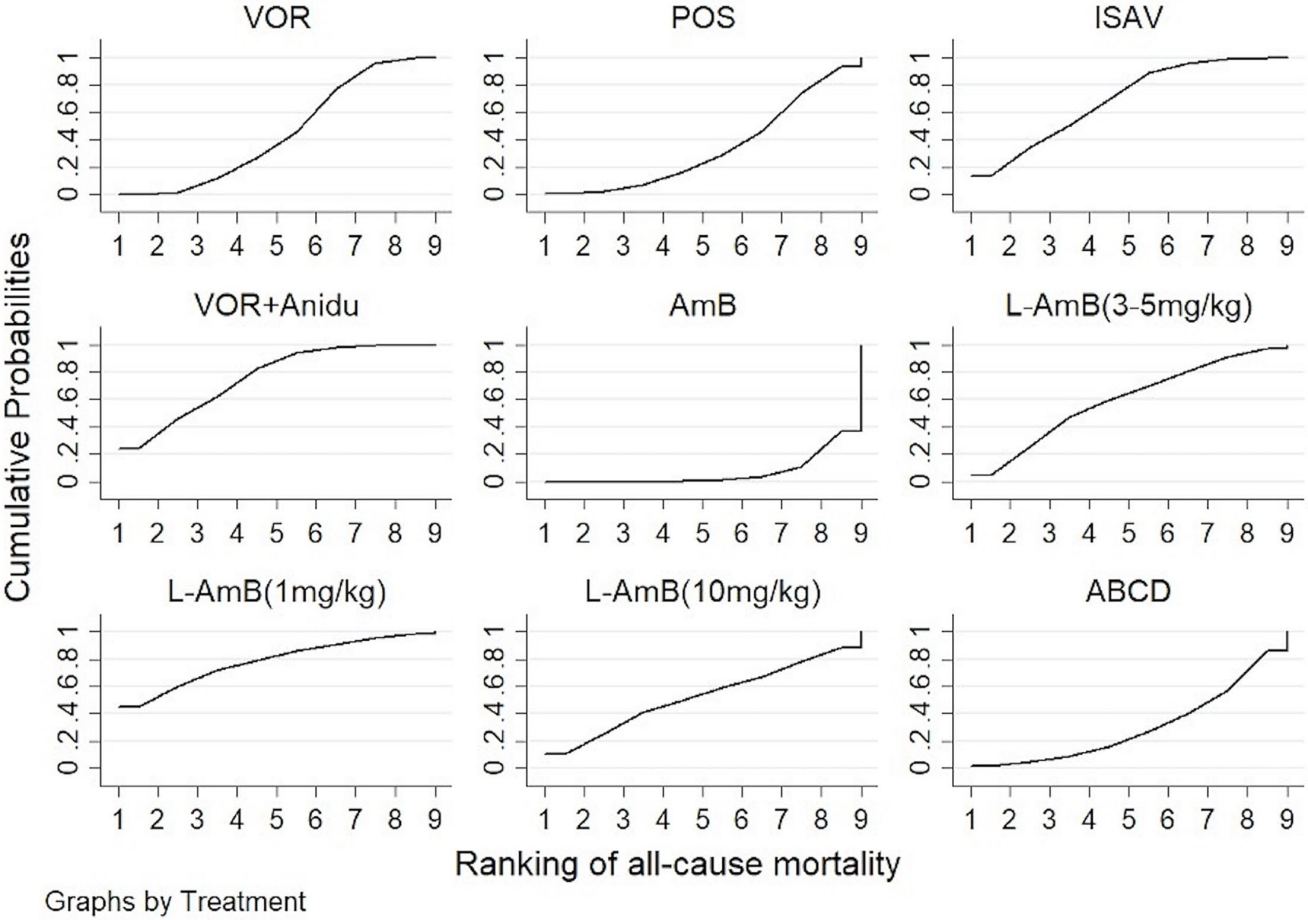
The ranking profile of comparable treatments on reducing all-cause mortality (ACM) for patients with invasive pulmonary aspergillosis (IPA). The X-axis represents ranking, and the Y-axis cumulative probabilities. (VOR, voriconazole; POS, Posaconazole; ISAV, isavuconazole; Anidu: anidulafungin; AmB, deoxycholate amphotericin B; L-AmB, liposomal amphotericin B; ABCD, amphotericin B colloidal dispersion).

Based on our analysis, the combination of voriconazole with anidulafungin, as well as isavuconazole and voriconazole alone, demonstrated superior safety compared to dAmB (Table 2). Compared to dAmB, the combination of voriconazole with anidulafungin (OR 0.33; 95% CI:0.16–0.69), isavuconazole (OR 0.38; 95% CI:0.18–0.78) and voriconazole alone (OR 0.52; 95% CI:0.30–0.88) exhibited lower odd ratios in ACM that may suggest potentially superior tolerability of treatment. However, the difference between posaconazole and dAmB in ACM was not found. According to the ranking profiles, L-AmB at a dosage of 1 mg/kg/d demonstrated the highest probability (SUCRA, 78.5%) for reducing ACM, followed by the combination of voriconazole and anidulafungin (SUCRA, 75.1%), and isavuconazole (SUCRA, 68.0%) (Figure 4; Supplementary Table S2).

**Table 2:** Pooled odds ratios (95% confidence intervals) for the number of all-cause deaths/the total number of patients included.

L-AmB(1 mg/kg)	1.25 (0.23,6.69)	1.41 (0.26,7.56)	1.46 (0.61,3.51)	1.64 (0.44,6.04)	1.94 (0.39,9.63)	2.26 (0.43,11.97)	2.55 (0.46,14.04)	3.76 (0.83,17.00)
0.80 (0.15,4.29)	VOR + Anidu	1.13 (0.55,2.30)	1.17 (0.28,4.91)	1.31 (0.23,7.39)	1.56 (0.94,2.57)	1.81 (0.92,3.58)	2.05 (0.70,6.02)	**3.01 (1.45,6.27)**
0.71 (0.13,3.80)	0.89 (0.43,1.81)	ISAV	1.04 (0.25,4.35)	1.16 (0.21,6.55)	1.38 (0.83,2.28)	1.61 (0.81,3.18)	1.81 (0.62,5.33)	**2.67 (1.28,5.56)**
0.68 (0.28,1.64)	0.85 (0.20,3.57)	0.96 (0.23,4.04)	L-AmB(3-5 mg/kg)	1.12 (0.42,2.95)	1.33 (0.35,5.07)	1.55 (0.37,6.38)	1.74 (0.40,7.53)	2.57 (0.75,8.78)
0.61 (0.17,2.26)	0.76 (0.14,4.30)	0.86 (0.15,4.86)	0.89 (0.34,2.36)	L-AmB(10 mg/kg)	1.19 (0.23,6.21)	1.38 (0.25,7.70)	1.56 (0.27,9.03)	2.30 (0.48,11.00)
0.51 (0.10,2.55)	0.64 (0.39,1.06)	0.73 (0.44,1.20)	0.75 (0.20,2.88)	0.84 (0.16,4.41)	VOR	1.17 (0.74,1.84)	1.31 (0.51,3.41)	**1.93 (1.13,3.30)**
0.44 (0.08,2.34)	0.55 (0.28,1.09)	0.62 (0.31,1.23)	0.65 (0.16,2.67)	0.72 (0.13,4.03)	0.86 (0.54,1.36)	POS	1.13 (0.39,3.25)	1.66 (0.82,3.35)
0.39 (0.07,2.15)	0.49 (0.17,1.44)	0.55 (0.19,1.63)	0.57 (0.13,2.48)	0.64 (0.11,3.71)	0.76 (0.29,1.98)	0.89 (0.31,2.55)	ABCD	1.47 (0.67,3.25)
0.27 (0.06,1.20)	**0.33 (0.16,0.69)**	**0.38 (0.18,0.78)**	0.39 (0.11,1.33)	0.44 (0.09,2.09)	**0.52 (0.30,0.88)**	0.60 (0.30,1.22)	0.68 (0.31,1.50)	dAmB

Data in each cell are odd ratios (95% confidence intervals) for comparing row-defining treatment versus column-defining treatment. Odds ratios less than one favor row-defining treatment. The results in bold are significant. VOR, voriconazole; POS, Posaconazole; ISAV, isavuconazole; Anidu: anidulafungin; dAmB, deoxycholate amphotericin B; L-AmB, liposomal amphotericin B; ABCD, amphotericin B colloidal dispersion.

### 3.4 Adverse events

Due to the limited availability of comparable information from included trials, we conducted a comparison of treatment-emergent adverse event rates by system organ class among voriconazole, posaconazole, isavuconazole, and the combination of voriconazole with anidulafungin (Supplementary Tables S3, S4). We found that posaconazole may exhibit a higher frequency of treatment-emergent adverse events, while isavuconazole showed comparatively better safety profiles.

Of the four anti-fungal agents, posaconazole showed the highest association with metabolism and nutrition disorders (SUCRA, 95.9%), followed by the combination of voriconazole with anidulafungin (60.2%), voriconazole alone (35.4%), and isavuconazole (8.5%). Regarding skin and subcutaneous tissue disorders, posaconazole was associated with the highest rates of adverse events (SUCRA, 69.7%), while isavuconazole showed the lowest association (SUCRA, 3.8%).

Among the three triazoles, namely voriconazole, posaconazole, and isavuconazole, posaconazole displayed the highest incidence of hepatobiliary disorders (SUCRA, 97.7%), while isavuconazole had a relatively low probability of 0.4% for hepatobiliary disorders. Likewise, posaconazole had the highest incidence of renal and urinary disorders (SUCRA, 98.4%), followed by voriconazole (30.7%) and isavuconazole (20.9%). Finally, we indirectly compared the rates of drug-related adverse events between posaconazole and isavuconazole. The results showed no significant difference between them (Supplementary Table S5).

## 4 Discussion

In our meta-analysis, we reviewed eight studies, which included 1,431 patients randomly assigned to different antifungal drugs in the initial treatment of IPA. We found second-generation triazole antifungal drugs, including voriconazole, isavuconazole, and posaconazole, exhibited significantly superior overall response compared to dAmB and ABCD. However, there was no difference between triazole antifungal drugs and L-AmB. In terms of safety, isavuconazole is likely to be better than voriconazole and posaconazole in the first-line treatment of IPA. The combination of voriconazole with anidulafungin may have a better safety profile than dAmB.

At present, voriconazole with the option of substituting dAmB is recommended as the first-line treatment for IPA patients by guidelines due to the unfavorable safety profile of dAmB (Herbrecht et al., 2002; Herbrecht et al., 2015; Patterson et al., 2016; Tissot et al., 2017). ABCD, as a high-affinity lipid complex composed of dAmB and sodium cholesteryl sulfate, exhibited superior renal safety compared to dAmB (White et al., 1997; White et al., 1998). However, its use showed more infusion-related toxicities resulting in premature discontinuation in patients who received ABCD in the study (Bowden et al., 2002). Triazole antifungal drugs have become the primary treatment options for patients diagnosed with IPA based on their better safety and tolerability (Maertens et al., 2016; Maertens et al., 2021). The results of our analysis showed that all voriconazole, isavuconazole, and posaconazole significantly improved survival compared to dAmB and ABCD.

L-AmB is associated with less nephrotoxicity and fewer infusion-related reactions than dAmB and ABCD (Leenders et al., 1998). By the IDSA guidelines, L-AmB can be recognized as the initial treatment option and a salvage option for patients who are not candidates for voriconazole treatment (Karthaus, 2010; Patterson et al., 2016). The recommended initial dosage of L-AmB to treat IPA is 3-5 mg/kg per day. The previous RCTs reported that L-AmB at 10 mg/kg did not provide any additional clinical benefit than the standard dosing (Cornely et al., 2007; Cornely et al., 2011). In our analysis, we found no statistically significant difference between triazole antifungal drugs and L-AmB at low, standard, or high doses. The result is consistent with a previous meta-analysis of trials involving isavuconazole (Herbrecht et al., 2018). Our analysis showed the overall SUCRA rank was higher for voriconazole but the probability of being the best agent was highest for L-AmB (1 mg/Kg). However, SUCRA with high values may provide supportive evidence for treatment options, but not conclusive evidence (Wang and Carter, 2018). The uncertainty of SUCRA rankings was also affected by a small number of trials or limited sample size (Trinquart et al., 2016). Although L-AmB (1 mg/kg) may rank better than triazole drugs in the small number of all-cause deaths by Supplementary Table S2, we found the median duration of treatment with L-AmB at any doses was shorter than those for triazoles in reviewed studies. For patients who need to receive sustaining treatment, triazoles may be preferred over L-AmB. In addition, oral triazoles may be more available to outpatients than intravenous L-AmB.

Studies have revealed that isavuconazole and posaconazole both exhibited comparable efficacy to voriconazole (Maertens et al., 2016; Maertens et al., 2021), but there are differences in the safety and tolerability of different triazoles. The use of voriconazole may be associated with drug–drug interactions, pharmacokinetic variability, and adverse events. The primary treatment-associated adverse effects of voriconazole include hepatotoxicity, neurological and visual disturbances, phototoxic reactions, tachyarrhythmias, and a high risk of nonmelanoma skin cancer (Benitez and Carver, 2019). Among the three triazole antifungals, isavuconazole may show the best safety profile. We found that isavuconazole and voriconazole demonstrated significantly better safety compared to AmB, but isavuconazole had significantly lower rates of hepatobiliary disorders (9% vs. 16%, *p* = 0.016), eye disorders (15% vs. 27%, *p* = 0.002), and skin or subcutaneous tissue disorders (33% vs. 42%, *p* = 0.037) compared to voriconazole in the 2016 study (Maertens et al., 2016). Posaconazole efficacy as well as isavuconazole is less affected than voriconazole by cytochrome P450 3A4 (CYP3A4) (Townsend et al., 2017; Chen et al., 2020). However, posaconazole may display a higher incidence of hepatobiliary disorders and renal and urinary disorders than isavuconazole. Our results suggested that posaconazole ranked lower than isavuconazole in terms of reducing ACM. When choosing posaconazole over isavuconazole to treat IPA, the monitoring of liver function and electrolytes is required throughout the treatment period.

The comparison between combination therapy with antifungal agents and monotherapy remains uncertain despite preclinical studies both *in vitro* and *in vivo* consistently supporting the use of azoles in combination with echinocandins for the treatment of IPA (Singh et al., 2006; Vazquez, 2008). In this analysis, we found that combining voriconazole with anidulafungin may have an acceptable safety profile in the initial management of IPA. The combination of voriconazole with anidulafungin had a tendency to improve 6-week survival compared to voriconazole monotherapy, although the difference was not statistically significant (Marr et al., 2015). Our findings suggested that the ACM up until day 84 was significantly lower for the combination of anidulafungin and voriconazole compared to dAmB, indicating a potential safety advantage. However, we found that the combination therapy ranked lower than other triazole monotherapy in overall response. The findings were consistent with the earlier analysis (Liu et al., 2024). We also found that the combination therapy was associated with a high incidence of eye disorders and vascular disorders (Supplementary Tables S3, S4). Additionally, it is worth noting that the actual median duration of the combination therapy was 14 days (range, 14–29 days) in the 2015 study, which was shorter than those for the voriconazole monotherapy (Marr et al., 2015). Therefore, it is essential for doctors to make cautious decisions regarding the use of combination therapy for the first-line treatment of IPA, considering individualized assessments and carefully weighing the potential risks and benefits.

Our analysis has certain limitations. Firstly, there is a limited number of RCTs available for the treatment of IPA that meet our inclusion criteria. We have to rely only on direct comparisons to evaluate the efficacy of different treatments. Additionally, due to the limitations of the data we included, we were unable to perform more comprehensive analyses, such as subgroup analyses based on different patient conditions or age groups. Secondly, some studies included a portion of patients infected with other non-Aspergillus molds. The study comparing a high-loading dose of L-AmB with standard dosing included 97% of patients with invasive aspergillosis. In the study comparing isavuconazole and voriconazole, the isavuconazole group (approximately 13%) enrolled a relatively small percentage of patients with infections due to non-Aspergillus molds or other unidentified filamentous fungi, as well as the voriconazole group (approximately 17%). While the majority of patients included in each study had pulmonary infection, there were instances where some studies included patients without pulmonary infection. These could have potentially influenced the results of our analysis to some extent. Thirdly, the earlier study included in the analysis did not utilize the revised EORTC/MSG criteria published in 2008 for diagnosing invasive aspergillosis (IA) (Ellis et al., 1998). This discrepancy may introduce bias in the comparison results. In addition, we excluded a randomized pilot study exploring the combination of L-AmB and caspofungin for IPA, given the risk of bias in the estimation of relative efficacy due to the small number of patients enrolled in each group (*n* = 15) (Caillot et al., 2007). Notably, there is a lack of RCT reporting efficacy between triazole drugs and L-AmB or efficacy among new triazole drugs other than voriconazole. The findings of our analysis do provide some recommendations for future research endeavors. In the future, more high-quality randomized controlled trials are needed to compare the effects of different antifungal drugs on invasive pulmonary aspergillosis.

## 5 Conclusion

This network meta-analysis has provided an indirect comparison of the efficacy of various drugs for the first-line treatment of IPA. Our findings indicate that the second-generation triazole antifungal drugs might be comparable with L-AmB and associated with higher therapeutic efficacy than dAmB and ABCD in IPA treatment. The safety of isavuconazole is probably better than voriconazole and posaconazole. Combination therapy with voriconazole and anidulafungin may serve as an alternative option for IPA patients with limited drug tolerance.

## Data Availability

The original contributions presented in the study are included in the article/Supplementary material, further inquiries can be directed to the corresponding author.

## References

[ref1] Andes D. Azie N. Yang H. Harrington R. Kelley C. Tan R. D. . (2016). Drug-drug interaction associated with Mold-active Triazoles among hospitalized patients. Antimicrob. Agents Chemother. 60, 3398–3406. doi: 10.1128/aac.00054-16, PMID: 27001815 PMC4879403

[ref2] Benitez L. L. Carver P. L. (2019). Adverse effects associated with long-term Administration of Azole Antifungal Agents. Drugs 79, 833–853. doi: 10.1007/s40265-019-01127-8, PMID: 31093949

[ref3] Bowden R. Chandrasekar P. White M. H. Li X. Pietrelli L. Gurwith M. . (2002). A double-blind, randomized, controlled trial of amphotericin B colloidal dispersion versus amphotericin B for treatment of invasive aspergillosis in immunocompromised patients. Clin. Infect. Dis. 35, 359–366. doi: 10.1086/341401, PMID: 12145716

[ref4] Caillot D. Thiébaut A. Herbrecht R. de Botton S. Pigneux A. Bernard F. . (2007). Liposomal amphotericin B in combination with caspofungin for invasive aspergillosis in patients with hematologic malignancies: a randomized pilot study (Combistrat trial). Cancer 110, 2740–2746. doi: 10.1002/cncr.23109, PMID: 17941026

[ref5] Chen L. Krekels E. H. J. Verweij P. E. Buil J. B. Knibbe C. A. J. Brüggemann R. J. M. (2020). Pharmacokinetics and pharmacodynamics of Posaconazole. Drugs 80, 671–695. doi: 10.1007/s40265-020-01306-y, PMID: 32323222 PMC7183491

[ref6] Cornely O. A. Maertens J. Bresnik M. Ebrahimi R. Dellow E. Herbrecht R. . (2011). Efficacy outcomes in a randomised trial of liposomal amphotericin B based on revised EORTC/MSG 2008 definitions of invasive mould disease. Mycoses 54, e449–e455. doi: 10.1111/j.1439-0507.2010.01947.x 21039936

[ref7] Cornely O. A. Maertens J. Bresnik M. Ebrahimi R. Ullmann A. J. Bouza E. . (2007). Liposomal amphotericin B as initial therapy for invasive mold infection: a randomized trial comparing a high-loading dose regimen with standard dosing (AmBiLoad trial). Clin. Infect. Dis. 44, 1289–1297. doi: 10.1086/514341 17443465

[ref8] Ellis M. Spence D. de Pauw B. Meunier F. Marinus A. Collette L. . (1998). An EORTC international multicenter randomized trial (EORTC number 19923) comparing two dosages of liposomal amphotericin B for treatment of invasive aspergillosis. Clin. Infect. Dis. 27, 1406–1412. doi: 10.1086/515033, PMID: 9868651

[ref9] Hajjeh R. A. Warnock D. W. (2001). Counterpoint: invasive aspergillosis and the environment--rethinking our approach to prevention. Clin. Infect. Dis. 33, 1549–1552. doi: 10.1086/322970, PMID: 11568854

[ref10] Hamill R. J. (2013). Amphotericin B formulations: a comparative review of efficacy and toxicity. Drugs 73, 919–934. doi: 10.1007/s40265-013-0069-4, PMID: 23729001

[ref11] Herbrecht R. Denning D. W. Patterson T. F. Bennett J. E. Greene R. E. Oestmann J. W. . (2002). Voriconazole versus amphotericin B for primary therapy of invasive aspergillosis. N. Engl. J. Med. 347, 408–415. doi: 10.1056/NEJMoa020191, PMID: 12167683

[ref12] Herbrecht R. Kuessner D. Pooley N. Posthumus J. Escrig C. (2018). Systematic review and network meta-analysis of clinical outcomes associated with isavuconazole versus relevant comparators for patients with invasive aspergillosis. Curr. Med. Res. Opin. 34, 2187–2195. doi: 10.1080/03007995.2018.1502659, PMID: 30022696

[ref13] Herbrecht R. Patterson T. F. Slavin M. A. Marchetti O. Maertens J. Johnson E. M. . (2015). Application of the 2008 definitions for invasive fungal diseases to the trial comparing voriconazole versus amphotericin B for therapy of invasive aspergillosis: a collaborative study of the mycoses study group (MSG 05) and the European Organization for Research and Treatment of Cancer infectious diseases group. Clin. Infect. Dis. 60, 713–720. doi: 10.1093/cid/ciu911, PMID: 25414266

[ref14] Karthaus M. (2010). Guideline based treatment of invasive aspergillosis. Mycoses 53, 36–43. doi: 10.1111/j.1439-0507.2009.01840.x 20433655

[ref15] Lamoth F. Calandra T. (2022). Pulmonary aspergillosis: diagnosis and treatment. Eur. Respir. Rev. 31:220114. doi: 10.1183/16000617.0114-2022 36450372 PMC9724826

[ref16] Ledoux M. P. Herbrecht R. (2023). Invasive pulmonary Aspergillosis. J. Fungi 9:131. doi: 10.3390/jof9020131, PMID: 36836246 PMC9962768

[ref17] Leenders A. C. Daenen S. Jansen R. L. Hop W. C. Lowenberg B. Wijermans P. W. . (1998). Liposomal amphotericin B compared with amphotericin B deoxycholate in the treatment of documented and suspected neutropenia-associated invasive fungal infections. Br. J. Haematol. 103, 205–212. doi: 10.1046/j.1365-2141.1998.00944.x, PMID: 9792309

[ref18] Liu A. Xiong L. Wang L. Zhuang H. Gan X. Zou M. . (2024). Compare the efficacy of antifungal agents as primary therapy for invasive aspergillosis: a network meta-analysis. BMC Infect. Dis. 24:581. doi: 10.1186/s12879-024-09477-9, PMID: 38867163 PMC11170913

[ref19] Lu G. Ades A. E. (2004). Combination of direct and indirect evidence in mixed treatment comparisons. Stat. Med. 23, 3105–3124. doi: 10.1002/sim.1875 15449338

[ref20] Maertens J. A. Raad I. I. Marr K. A. Patterson T. F. Kontoyiannis D. P. Cornely O. A. . (2016). Isavuconazole versus voriconazole for primary treatment of invasive mould disease caused by aspergillus and other filamentous fungi (SECURE): a phase 3, randomised-controlled, non-inferiority trial. Lancet 387, 760–769. doi: 10.1016/s0140-6736(15)01159-9 26684607

[ref21] Maertens J. A. Rahav G. Lee D. G. Ponce-de-León A. Ramírez Sánchez I. C. Klimko N. . (2021). Posaconazole versus voriconazole for primary treatment of invasive aspergillosis: a phase 3, randomised, controlled, non-inferiority trial. Lancet 397, 499–509. doi: 10.1016/s0140-6736(21)00219-1, PMID: 33549194

[ref22] Marr K. A. Schlamm H. T. Herbrecht R. Rottinghaus S. T. Bow E. J. Cornely O. A. . (2015). Combination antifungal therapy for invasive aspergillosis: a randomized trial. Ann. Intern. Med. 162, 81–89. doi: 10.7326/m13-2508, PMID: 25599346

[ref23] Neofytos D. Avdic E. Magiorakos A. P. (2010). Clinical safety and tolerability issues in use of triazole derivatives in management of fungal infections. Drug Healthc Patient Saf. 2, 27–38. doi: 10.2147/dhps.s6321 21701616 PMC3108707

[ref24] Patterson T. F. Thompson G. R. 3rd Denning D. W. Fishman J. A. Hadley S. Herbrecht R. . (2016). Practice guidelines for the diagnosis and Management of Aspergillosis: 2016 update by the Infectious Diseases Society of America. Clin. Infect. Dis. 63, e1–e60. doi: 10.1093/cid/ciw326, PMID: 27365388 PMC4967602

[ref25] Rouse B. Chaimani A. Li T. (2017). Network meta-analysis: an introduction for clinicians. Intern. Emerg. Med. 12, 103–111. doi: 10.1007/s11739-016-1583-7, PMID: 27913917 PMC5247317

[ref26] Salanti G. Ades A. E. Ioannidis J. P. (2011). Graphical methods and numerical summaries for presenting results from multiple-treatment meta-analysis: an overview and tutorial. J. Clin. Epidemiol. 64, 163–171. doi: 10.1016/j.jclinepi.2010.03.016, PMID: 20688472

[ref27] Singh N. Limaye A. P. Forrest G. Safdar N. Muñoz P. Pursell K. . (2006). Combination of voriconazole and caspofungin as primary therapy for invasive aspergillosis in solid organ transplant recipients: a prospective, multicenter, observational study. Transplantation 81, 320–326. doi: 10.1097/01.tp.0000202421.94822.f7, PMID: 16477215

[ref28] Stevens D. A. Kan V. L. Judson M. A. Morrison V. A. Dummer S. Denning D. W. . (2000). Practice guidelines for diseases caused by aspergillus. Infectious Diseases Society of America. Clin. Infect. Dis. 30, 696–709. doi: 10.1086/313756 10770732

[ref29] Tiphine M. Letscher-Bru V. Herbrecht R. (1999). Amphotericin B and its new formulations: pharmacologic characteristics, clinical efficacy, and tolerability. Transpl. Infect. Dis. 1, 273–283. doi: 10.1034/j.1399-3062.1999.010406.x 11428998

[ref30] Tissot F. Agrawal S. Pagano L. Petrikkos G. Groll A. H. Skiada A. . (2017). ECIL-6 guidelines for the treatment of invasive candidiasis, aspergillosis and mucormycosis in leukemia and hematopoietic stem cell transplant patients. Haematologica 102, 433–444. doi: 10.3324/haematol.2016.152900, PMID: 28011902 PMC5394968

[ref31] Townsend R. Dietz A. Hale C. Akhtar S. Kowalski D. Lademacher C. . (2017). Pharmacokinetic evaluation of CYP3A4-mediated drug-drug interactions of Isavuconazole with rifampin, ketoconazole, midazolam, and Ethinyl estradiol/Norethindrone in healthy adults. Clin. Pharmacol. Drug Dev. 6, 44–53. doi: 10.1002/cpdd.285, PMID: 27273461 PMC5298035

[ref32] Trinquart L. Attiche N. Bafeta A. Porcher R. Ravaud P. (2016). Uncertainty in treatment rankings: reanalysis of network Meta-analyses of randomized trials. Ann. Intern. Med. 164, 666–673. doi: 10.7326/m15-2521 27089537

[ref33] Ullmann A. J. Aguado J. M. Arikan-Akdagli S. Denning D. W. Groll A. H. Lagrou K. . (2018). Diagnosis and management of aspergillus diseases: executive summary of the 2017 ESCMID-ECMM-ERS guideline. Clin. Microbiol. Infect. 24, e1–e38. doi: 10.1016/j.cmi.2018.01.002, PMID: 29544767

[ref34] Vazquez J. A. (2008). Clinical practice: combination antifungal therapy for mold infections: much ado about nothing? Clin. Infect. Dis. 46, 1889–1901. doi: 10.1086/588475 18466092

[ref35] Wang Z. Carter R. E. (2018). Ranking of the most effective treatments for cardiovascular disease using SUCRA: is it as sweet as it appears? Eur. J. Prev. Cardiol. 25, 842–843. doi: 10.1177/2047487318767199, PMID: 29569939

[ref36] White M. H. Anaissie E. J. Kusne S. Wingard J. R. Hiemenz J. W. Cantor A. . (1997). Amphotericin B colloidal dispersion vs. amphotericin B as therapy for invasive aspergillosis. Clin. Infect. Dis. 24, 635–642, PMID: 9145737

[ref37] White M. H. Bowden R. A. Sandler E. S. Graham M. L. Noskin G. A. Wingard J. R. . (1998). Randomized, double-blind clinical trial of amphotericin B colloidal dispersion vs. amphotericin B in the empirical treatment of fever and neutropenia. Clin. Infect. Dis. 27, 296–302. doi: 10.1086/514672, PMID: 9709879

