# Supplementary material for: Comparison of antifungal drugs in the treatment of invasive pulmonary aspergillosis: a systematic review and network meta-analysis
Source: Front Microbiol. 2024 Dec 2;15:1504826. doi: 10.3389/fmicb.2024.1504826 (PMC11648418; doi:10.3389/fmicb.2024.1504826)
Supplement: Supplementary file 1 [file Table_1.docx]

**Comparison of Antifungal Drugs in the Treatment of Invasive Pulmonary Aspergillosis: A Systematic Review and Network Meta-analysis**

**Additional files**

**Supplementary Table 1. Ranking probabilities (%) and Mean Ranks for different anti-fungal drugs with the favorable overall response for IPA**

|  | VOR | POS | ISAV | VOR+Anidu | AmB | L-AmB  (3-5mg/kg) | L-AmB  (1mg/kg) | L-AmB  (10mg/kg) | ABCD |
| --- | --- | --- | --- | --- | --- | --- | --- | --- | --- |
| Best | 18.8 | 8.6 | 19.3 | 1.1 | 0 | 0.3 | 39.9 | 12.0 | 0 |
| 2^nd^ | 31.5 | 13.3 | 19.7 | 2.8 | 0 | 5.6 | 12.1 | 14.9 | 0 |
| 3^rd^ | 24.0 | 22.8 | 19.4 | 7.8 | 0.1 | 10.5 | 8.0 | 7.3 | 0.1 |
| 4^th^ | 16.0 | 20.7 | 17.9 | 19.6 | 0.2 | 6.5 | 10.7 | 8.1 | 0.4 |
| 5^th^ | 8.0 | 16.1 | 12.0 | 20.4 | 3.3 | 11.7 | 14.9 | 12.0 | 1.5 |
| 6^th^ | 1.7 | 12.9 | 7.8 | 18.3 | 9.4 | 23.9 | 7.8 | 15.4 | 2.9 |
| 7^th^ | 0.1 | 5.3 | 3.6 | 25.2 | 16.4 | 25.5 | 4.1 | 13.8 | 5.9 |
| 8^th^ | 0 | 0.4 | 0.2 | 3.9 | 60.6 | 11.1 | 1.5 | 9.6 | 12.8 |
| Worst | 0 | 0 | 0,1 | 0.9 | 10.1 | 4.9 | 0.8 | 6.8 | 76.3 |
| Mean rank | 2.7 | 3.8 | 3.2 | 5.4 | 7.6 | 5.8 | 3.0 | 4.8 | 8.6 |
| SUCRA | 79.0 | 64.5 | 72.1 | 45.5 | 17.0 | 39.7 | 74.9 | 52.1 | 5.3 |

Abbreviations: IPA, invasive pulmonary aspergillosis; VOR, voriconazole; POS, Posaconazole; ISAV, isavuconazole; Anidu: anidulafungin; AmB, deoxycholate amphotericin B; L-AmB, liposomal amphotericin B; ABCD, amphotericin B colloidal dispersion; SUCRA, surface under the cumulative ranking curve.

**Supplementary Table 2. Ranking probabilities (%) and Mean Ranks for different anti-fungal drugs with the small number of all-cause deaths for IPA**

|  | VOR | POS | ISAV | VOR+Anidu | AmB | L-AmB  (3-5mg/kg) | L-AmB  (1mg/kg) | L-AmB  (10mg/kg) | ABCD |
| --- | --- | --- | --- | --- | --- | --- | --- | --- | --- |
| Best | 0.1 | 0.5 | 13.3 | 22.5 | 0 | 4.4 | 44.6 | 12.8 | 1.8 |
| 2^nd^ | 1.5 | 1.8 | 19.3 | 22.7 | 0 | 21.4 | 16.3 | 14.3 | 2.7 |
| 3^rd^ | 9.6 | 5.0 | 16.2 | 15.7 | 0 | 21.7 | 11.5 | 15.6 | 4.8 |
| 4^th^ | 14.4 | 9.2 | 19.6 | 20.0 | 0 | 13.2 | 6.8 | 9.1 | 7.6 |
| 5^th^ | 17.2 | 12.7 | 19.2 | 13.2 | 0.7 | 10.1 | 6.4 | 9.4 | 11.1 |
| 6^th^ | 32.9 | 16.6 | 7.5 | 3.8 | 2.8 | 11.0 | 5.6 | 7.4 | 12.4 |
| 7^th^ | 20.0 | 27.6 | 3.3 | 1.7 | 6.4 | 10.2 | 4.1 | 10.4 | 16.3 |
| 8^th^ | 4.1 | 20.4 | 1.4 | 0.5 | 27.1 | 5.9 | 3.1 | 9.1 | 28.4 |
| Worst | 0.2 | 6.2 | 0.1 | 0 | 63.0 | 2.0 | 1.5 | 12.0 | 14.9 |
| Mean rank | 5.5 | 6.3 | 3.6 | 3.0 | 8.5 | 4.2 | 2.7 | 4.7 | 6.6 |
| SUCRA | 44.1 | 33.6 | 68.0 | 75.1 | 6.4 | 60.1 | 78.5 | 53.8 | 30.4 |

Abbreviations: IPA, invasive pulmonary aspergillosis; VOR, voriconazole; POS, Posaconazole; ISAV, isavuconazole; Anidu: anidulafungin; AmB, deoxycholate amphotericin B; L-AmB, liposomal amphotericin B; ABCD, amphotericin B colloidal dispersion; SUCRA, surface under the cumulative ranking curve.

**Supplementary Table 3 Comparisons of treatment-emergent adverse events by system organ class among voriconazole, posaconazole, isavuconazole and voriconazole with anidulafungin in combination. (Pooled odds ratios for each available comparison on each specific adverse event)**

| Treatment-emergent adverse events | VOR vs | | | POS vs | | ISAV vs |
| --- | --- | --- | --- | --- | --- | --- |
|  | **POS** | **ISA** | **VOR+Anidu** | **ISAV** | **VOR+Anidu** | **VOR+Anidu** |
| Blood and lymphatic system disorders | 0.86 (0.60,1.22) | 1.08 (0.75,1.57) | 1.25 (0.77,2.04) | 1.27 (0.76,2.11) | 1.46 (0.80,2.67) | 1.16 (0.63,2.14) |
| Cardiac disorders | 1.13 (0.74,1.75) | 1.40 (0.90,2.18) | 1.07 (0.67,1.70) | 1.24 (0.67,2.30) | 0.94 (0.50,1.78) | 0.76 (0.40,1.45) |
| Ear and labyrinth disorders | 1.24 (0.50,3.03) | 0.92 (0.42,1.99) | / | 0.74 (0.23,2.43) | / | / |
| Eye disorders | 1.45 (0.91,2.32) | **2.03 (1.31,3.15)** | 1.26 (0.82,1.95) | 1.40 (0.74,2.66) | 0.87 (0.46,1.65) | 0.62 (0.34,1.15) |
| Gastrointestinal disorders | 1.02 (0.74,1.42) | 1.09 (0.75,1.58) | 1.08 (0.74,1.57) | 1.06 (0.65,1.75) | 1.05 (0.64,1.73) | 0.99 (0.59,1.68) |
| General disorders and administration site | 0.78 (0.56,1.09) | 0.92 (0.65,1.31) | 1.15 (0.80,1.67) | 1.18 (0.73,1.90) | 1.47 (0.90,2.41) | 1.25 (0.75,2.08) |
| Hepatobiliary disorders | 0.64 (0.38,1.08) | **1.97 (1.15,3.38)** | / | **3.10 (1.46,6.58)** | / | / |
| Immune system disorders | 0.95 (0.50,1.80) | 1.27 (0.68,2.34) | / | 1.33 (0.55,3.22) | / | / |
| Infections and infestations | 0.92 (0.65,1.30) | 1.08 (0.76,1.54) | 0.91 (0.63,1.32) | 1.17 (0.72,1.92) | 0.99 (0.60,1.64) | 0.85 (0.51,1.41) |
| Injury, poisoning, and procedural | 1.00 (0.63,1.61) | 1.20 (0.73,1.98) | 0.59 (0.34,1.05) | 1.20 (0.60,2.38) | 0.59 (0.28,1.24) | 0.49 (0.23,1.05) |
| Investigations | 0.88 (0.64,1.23) | 1.19 (0.83,1.71) | 0.81 (0.55,1.20) | 1.35 (0.82,2.20) | 0.92 (0.55,1.53) | 0.68 (0.40,1.16) |
| Metabolism and nutrition disorders | **0.63 (0.45,0.88)** | 1.21 (0.85,1.71) | 0.86 (0.59,1.25) | **1.92 (1.19,3.11)** | 1.36 (0.82,2.25) | 0.71 (0.42,1.19) |
| Musculoskeletal and connective tissue | 0.80 (0.54,1.19) | 1.15 (0.79,1.69) | 0.86 (0.55,1.36) | 1.44 (0.83,2.50) | 1.07 (0.59,1.96) | 0.75 (0.41,1.35) |
| Neoplasms benign, malignant, and unspecified (including cysts and polyps) | 0.97 (0.57,1.64) | 1.70 (0.94,3.10) | / | 1.76 (0.79,3.90) | / | / |
| Nervous system disorders | 0.76 (0.53,1.08) | 0.89 (0.62,1.28) | 0.95 (0.63,1.43) | 1.18 (0.71,1.96) | 1.25 (0.73,2.15) | 1.06 (0.62,1.83) |
| Psychiatric disorders | 1.28 (0.86,1.90) | 1.33 (0.91,1.94) | 0.94 (0.63,1.38) | 1.04 (0.60,1.79) | 0.73 (0.42,1.27) | 0.70 (0.41,1.21) |
| Renal and urinary disorders | **0.60 (0.40,0.90)** | 1.06 (0.70,1.61) | / | 1.77 (0.99,3.18) | / | / |
| Reproductive system and breast disorders | 1.12 (0.45,2.80) | 1.64 (0.67,4.04) | / | 1.47 (0.41,5.30) | / | / |
| Skin and subcutaneous tissue disorders | 0.94 (0.65,1.35) | **1.47 (1.03,2.10)** | 0.94 (0.64,1.38) | 1.56 (0.94,2.61) | 1.00 (0.59,1.70) | 0.64 (0.38,1.08) |
| Vascular disorders | 1.03 (0.69,1.54) | 1.20 (0.82,1.76) | **0.63 (0.42,0.95)** | 1.17 (0.67,2.04) | 0.61 (0.34,1.09) | **0.52 (0.30,0.92)** |

Abbreviations: VOR, voriconazole; POS, Posaconazole; ISAV, isavuconazole; Anidu: anidulafungin. Significant values are in bold and colored in gray.

**Supplementary Table 4 SUCRA Values and Mean Rank for rates of treatment-emergent adverse events by system organ class among voriconazole, posaconazole, isavuconazole and voriconazole with anidulafungin in combination.**

| Treatment-emergent adverse events | VOR | | POS | | ISAV | | VOR+Anidu | |
| --- | --- | --- | --- | --- | --- | --- | --- | --- |
|  | SUCRA, % | Mean rank | SUCRA, % | Mean rank | SUCRA, % | Mean rank | SUCRA, % | Mean rank |
| Blood and lymphatic system disorders | 55.9 | 2.3 | 83.6 | 1.5 | 39.8 | 2.8 | 20.6 | 7.2 |
| Cardiac disorders | 75.3 | 1.7 | 48.9 | 2.5 | 17.4 | 3.5 | 58.4 | 2.2 |
| Ear and labyrinth disorders | 55.4 | 1.9 | 31.0 | 2.4 | 63.6 | 1.7 | / | / |
| Eye disorders | 92.8 | **1.2** | 41.4 | 2.8 | 7.1 | 3.8 | 58.6 | 2.2 |
| Gastrointestinal disorders | 63.0 | 2.1 | 54.4 | 2.4 | 39.7 | 2.8 | 42.8 | 2.7 |
| General disorders and administration site | 39.4 | 2.8 | 87.1 | 1.4 | 57.8 | 2.3 | 15.7 | 3.5 |
| Hepatobiliary disorders | 51.8 | 2.0 | 97.7 | **1.0** | 0.4 | 3.0 | / | / |
| Immune system disorders | 61.5 | 1.8 | 63.7 | 1.7 | 24.9 | 2.5 | / | / |
| Infections and infestations | 43.4 | 2.7 | 63.5 | 2.1 | 28.0 | 3.2 | 65.1 | 2.0 |
| Injury, poisoning, and procedural | 44.1 | 2.7 | 43.1 | 2.7 | 17.8 | 3.5 | 94.9 | 1.2 |
| Investigations | 40.1 | 2.8 | 66.8 | 2.0 | 12.8 | 3.6 | 80.3 | 1.6 |
| Metabolism and nutrition disorders | 35.4 | 2.9 | 95.9 | **1.1** | 8.5 | 3.7 | 60.2 | 2.2 |
| Musculoskeletal and connective tissue | 38.8 | 2.8 | 78.3 | 1.7 | 16.4 | 3.5 | 66.4 | 2.0 |
| Neoplasms benign, malignant, and unspecified (including cysts and polyps) | 70.6 | 1.6 | 73.3 | 1.5 | 6.1 | 2.9 | / | / |
| Nervous system disorders | 24.4 | 3.3 | 82.8 | 1.5 | 52.4 | 2.4 | 40.4 | 2.8 |
| Psychiatric disorders | 72.7 | 1.8 | 26.0 | 3.2 | 21.0 | 3.4 | 80.3 | 1.6 |
| Renal and urinary disorders | 30.7 | 2.4 | 98.4 | **1.0** | 20.9 | 2.6 | / | / |
| Reproductive system and breast disorders | 73.4 | 1.5 | 56.3 | 1.9 | 20.3 | 2.6 | / | / |
| Skin and subcutaneous tissue disorders | 57.0 | 2.3 | 69.7 | **1.9** | 3.8 | 3.9 | 69.5 | 1.9 |
| Vascular disorders | 45.9 | 2.6 | 40.8 | 2.8 | 15.7 | 3.5 | 97.6 | **1.1** |

Abbreviations: VOR, voriconazole; POS, Posaconazole; ISAV, isavuconazole; Anidu: anidulafungin. Each ranking first is in bold.

**Supplementary Table 5 Comparisons of rates of drug-related adverse events among voriconazole, Posaconazole and isavuconazole. (Pooled odds ratios for each treatment on incidences of drug-related adverse events)**

| VOR | 0.64 (0.45,0.90) | 0.49 (0.35,0.70) |
| --- | --- | --- |
| **1.57 (1.11,2.22)** | POS | 0.78 (0.47,1.27) |
| **2.02 (1.43,2.87)** | 1.29 (0.79,2.11) | ISAV |

Abbreviations: VOR, voriconazole; POS, Posaconazole; ISAV, isavuconazole. Significant values are in bold.

**Supplementary Table 6** Pooled estimates of the sensitivity analysis **excluding the study^19^: pooled odds ratios (95% credible intervals) for the number with favorable response/the total number of patients included**

| VOR | 0.94 (0.57,1.54) | 0.85 (0.55,1.32) | 0.64 (0.39,1.05) | 0.57 (0.14,2.39) | 0.52 (0.17,1.62) | **0.35 (0.20,0.60)** | **0.23 (0.09,0.59)** |
| --- | --- | --- | --- | --- | --- | --- | --- |
| 1.07 (0.65,1.75) | ISAV | 0.91 (0.47,1.76) | 0.68 (0.34,1.38) | 0.61 (0.13,2.77) | 0.55 (0.16,1.92) | **0.37 (0.18,0.77)** | **0.24 (0.08,0.71)** |
| 1.17 (0.76,1.80) | 1.10 (0.57,2.12) | POS | 0.75 (0.39,1.44) | 0.66 (0.15,2.98) | 0.61 (0.18,2.05) | **0.40 (0.20,0.81)** | **0.26 (0.09,0.75)** |
| 1.56 (0.95,2.54) | 1.46 (0.73,2.93) | 1.33 (0.69,2.56) | VOR+Anidu | 0.88 (0.19,4.03) | 0.81 (0.24,2.79) | 0.54 (0.26,1.12) | 0.35 (0.12,1.03) |
| 1.76 (0.42,7.41) | 1.65 (0.36,7.55) | 1.51 (0.34,6.75) | 1.13 (0.25,5.16) | L-AmB(10mg/kg) | 0.92 (0.38,2.20) | 0.61 (0.16,2.29) | 0.40 (0.09,1.86) |
| 1.92 (0.62,5.99) | 1.80 (0.52,6.23) | 1.64 (0.49,5.54) | 1.23 (0.36,4.25) | 1.09 (0.45,2.62) | L-AmB(3-5mg/kg) | 0.66 (0.25,1.79) | 0.43 (0.12,1.54) |
| **2.90 (1.67,5.02)** | **2.72 (1.30,5.70)** | **2.48 (1.23,4.98)** | 1.86 (0.89,3.88) | 1.64 (0.44,6.20) | 1.51 (0.56,4.08) | AmB | 0.66 (0.30,1.43) |
| **4.42 (1.70,11.47)** | **4.15(1.42,12.15)** | **3.78 (1.33,10.76)** | 2.84 (0.97,8.29) | 2.51 (0.54,11.69) | 2.30 (0.65,8.14) | 1.53 (0.70,3.33) | ABCD |

Data in each cell are odd ratios (95% credible intervals) for the comparison of row-defining treatment versus column-defining treatment. Odds ratios more than 1 favor row-defining treatment. The results in bold are significant. Abbreviations: VOR, voriconazole; POS, Posaconazole; ISAV, isavuconazole; Anidu: anidulafungin; AmB, deoxycholate amphotericin B; L-AmB, liposomal amphotericin B; ABCD, amphotericin B colloidal dispersion.

**Supplementary Table 7** Pooled estimates of the sensitivity analysis **excluding the study^19^: pooled odds ratios (95% credible intervals) for the number with all-cause deaths/the total number of patients included**

| VOR+Anidu | 1.13 (0.55,2.30) | 1.36 (0.37,5.05) | 1.56 (0.94,2.57) | 1.81 (0.92,3.58) | 1.86 (0.39,8.98) | 2.05 (0.70,6.02) | 3.01 (1.45,6.27) |
| --- | --- | --- | --- | --- | --- | --- | --- |
| 0.89 (0.43,1.81) | ISAV | 1.21 (0.32,4.48) | 1.38 (0.83,2.28) | 1.61 (0.81,3.18) | 1.65 (0.34,7.96) | 1.81 (0.62,5.33) | 2.67 (1.28,5.56) |
| 0.73 (0.20,2.72) | 0.83 (0.22,3.08) | L-AmB(3-5mg/kg) | 1.14 (0.34,3.84) | 1.33 (0.37,4.86) | 1.37 (0.57,3.26) | 1.50 (0.39,5.76) | 2.21 (0.75,6.55) |
| 0.64 (0.39,1.06) | 0.73 (0.44,1.20) | 0.87 (0.26,2.94) | VOR | 1.17 (0.74,1.84) | 1.20 (0.27,5.31) | 1.31 (0.51,3.41) | 1.93 (1.13,3.30) |
| 0.55 (0.28,1.09) | 0.62 (0.31,1.23) | 0.75 (0.21,2.74) | 0.86 (0.54,1.36) | POS | 1.03 (0.22,4.88) | 1.13 (0.39,3.25) | 1.66 (0.82,3.35) |
| 0.54 (0.11,2.59) | 0.61 (0.13,2.92) | 0.73 (0.31,1.74) | 0.84 (0.19,3.71) | 0.97 (0.20,4.63) | L-AmB(10mg/kg) | 1.10 (0.22,5.44) | 1.62 (0.40,6.49) |
| 0.49 (0.17,1.44) | 0.55 (0.19,1.63) | 0.67 (0.17,2.55) | 0.76 (0.29,1.98) | 0.89 (0.31,2.55) | 0.91 (0.18,4.51) | ABCD | 1.47 (0.67,3.25) |
| **0.33 (0.16,0.69)** | **0.38 (0.18,0.78)** | 0.45 (0.15,1.34) | **0.52 (0.30,0.88)** | 0.60 (0.30,1.22) | 0.62 (0.15,2.49) | 0.68 (0.31,1.50) | AmB |

Data in each cell are odd ratios (95% credible intervals) for the comparison of row-defining treatment versus column-defining treatment. Odds ratios less than 1 favor row-defining treatment. The results in bold are significant. Abbreviations: VOR, voriconazole; POS, Posaconazole; ISAV, isavuconazole; Anidu: anidulafungin; AmB, deoxycholate amphotericin B; L-AmB, liposomal amphotericin B; ABCD, amphotericin B colloidal dispersion.

**Supplementary Table 8 Literature search strategy**

| Search (("Invasive Pulmonary Aspergillosis"[MeSH Terms] OR ("aspergillosis invasive pulmonary"[Title/Abstract] OR (("pulmonary aspergillosis"[MeSH Terms] OR ("Pulmonary"[All Fields] AND "Aspergillosis"[All Fields]) OR "pulmonary aspergillosis"[All Fields] OR ("Pulmonary"[All Fields] AND "aspergilloses"[All Fields]) OR "pulmonary aspergilloses"[All Fields]) AND "Invasive"[Title/Abstract]) OR "pulmonary aspergillosis invasive"[Title/Abstract] OR "pulmonary aspergillosis invasive"[Title/Abstract] OR "pulmonary aspergillosis invasive"[Title/Abstract] OR "chronic necrotizing pulmonary aspergillosis"[Title/Abstract] OR "allergic bronchopulmonary mycosis"[Title/Abstract] OR "allergic bronchopulmonary mycoses"[Title/Abstract] OR "bronchopulmonary mycoses allergic"[Title/Abstract] OR "bronchopulmonary mycosis allergic"[Title/Abstract] OR (("Mycoses"[MeSH Terms] OR "Mycoses"[All Fields] OR "trehalose"[MeSH Terms] OR "trehalose"[All Fields] OR "mycose"[All Fields]) AND "allergic bronchopulmonary"[Title/Abstract]) OR (("Mycoses"[MeSH Terms] OR "Mycoses"[All Fields] OR "Mycosis"[All Fields]) AND "allergic bronchopulmonary"[Title/Abstract]) OR "IA"[All Fields] OR "IPA"[All Fields])) AND ("Therapeutics"[MeSH Terms] OR ("Therapeutic"[Title/Abstract] OR "Therapy"[Title/Abstract] OR "Therapies"[Title/Abstract] OR "Treatment"[Title/Abstract] OR "Treatments"[Title/Abstract])) AND ("randomized controlled trial"[Publication Type] OR "controlled clinical trial"[Publication Type] OR "randomized"[Title/Abstract] OR "placebo"[Title/Abstract] OR "clinical trials as topic"[MeSH Terms] OR "randomly"[Title/Abstract] OR "trial"[Title])) NOT ("animals"[MeSH Terms] NOT "humans"[MeSH Terms]) |
| --- |


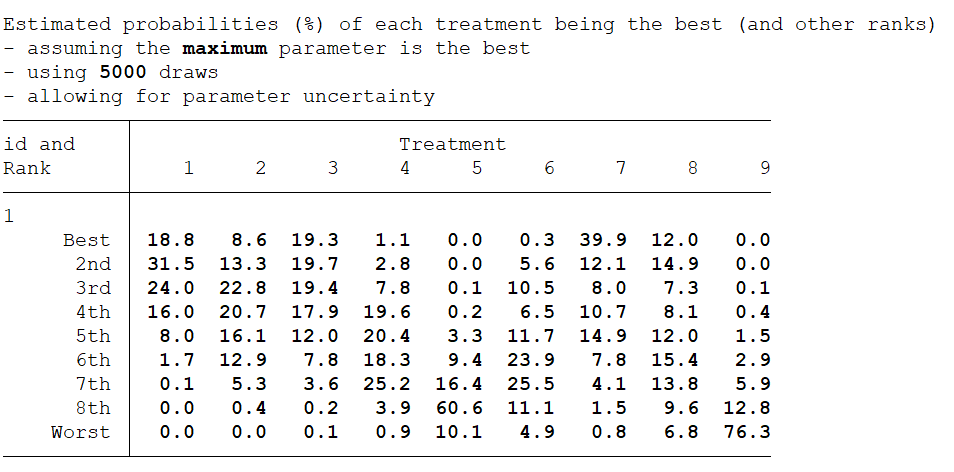

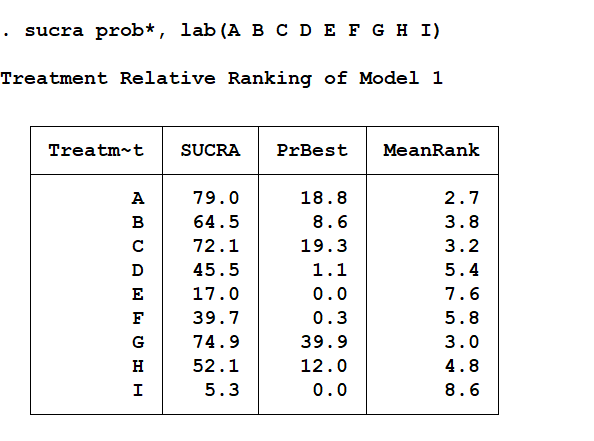


**Supplementary Figure 1. The ranking program and SUCRA value in overall response by** **using Stata statistical software version 14.2.** The number 1 and the letter A represent voriconazole, the number 2 and the letter B represent posaconazole, the number 3 and the letter C represent isavuconazole, the number 4 and the letter D represent the combination of voriconazole and anidufungin, the number 5 and the letter E represent dAmB, the number 6 and the letter F represent L-AmB (3-5mg/kg), the number 7 and the letter G represent L-AmB (1mg/kg), the number 8 and the letter H represent L-AmB (10mg/kg), the number 9 and the letter I represent ABCD. dAmB, deoxycholate amphotericin B; L-AmB, liposomal amphotericin B; ABCD, amphotericin B colloidal dispersion.


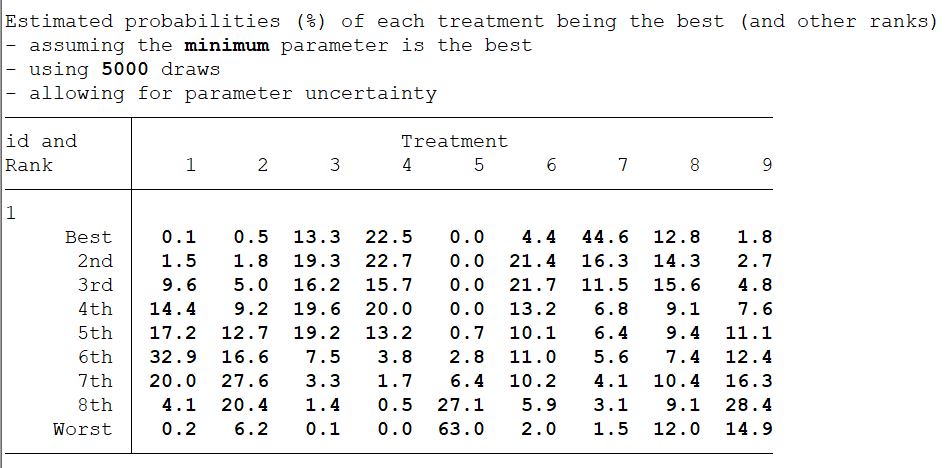

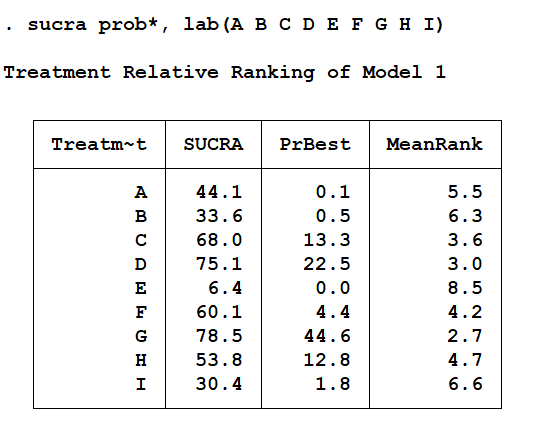


**Supplementary Figure 2. The ranking program and SUCRA value in all-cause mortality (ACM) by** **using Stata statistical software version 14.2.** The number 1 and the letter A represent voriconazole, the number 2 and the letter B represent posaconazole, the number 3 and the letter C represent isavuconazole, the number 4 and the letter D represent the combination of voriconazole and anidufungin, the number 5 and the letter E represent dAmB, the number 6 and the letter F represent L-AmB (3-5mg/kg), the number 7 and the letter G represent L-AmB (1mg/kg), the number 8 and the letter H represent L-AmB (10mg/kg), the number 9 and the letter I represent ABCD. dAmB, deoxycholate amphotericin B; L-AmB, liposomal amphotericin B; ABCD, amphotericin B colloidal dispersion.


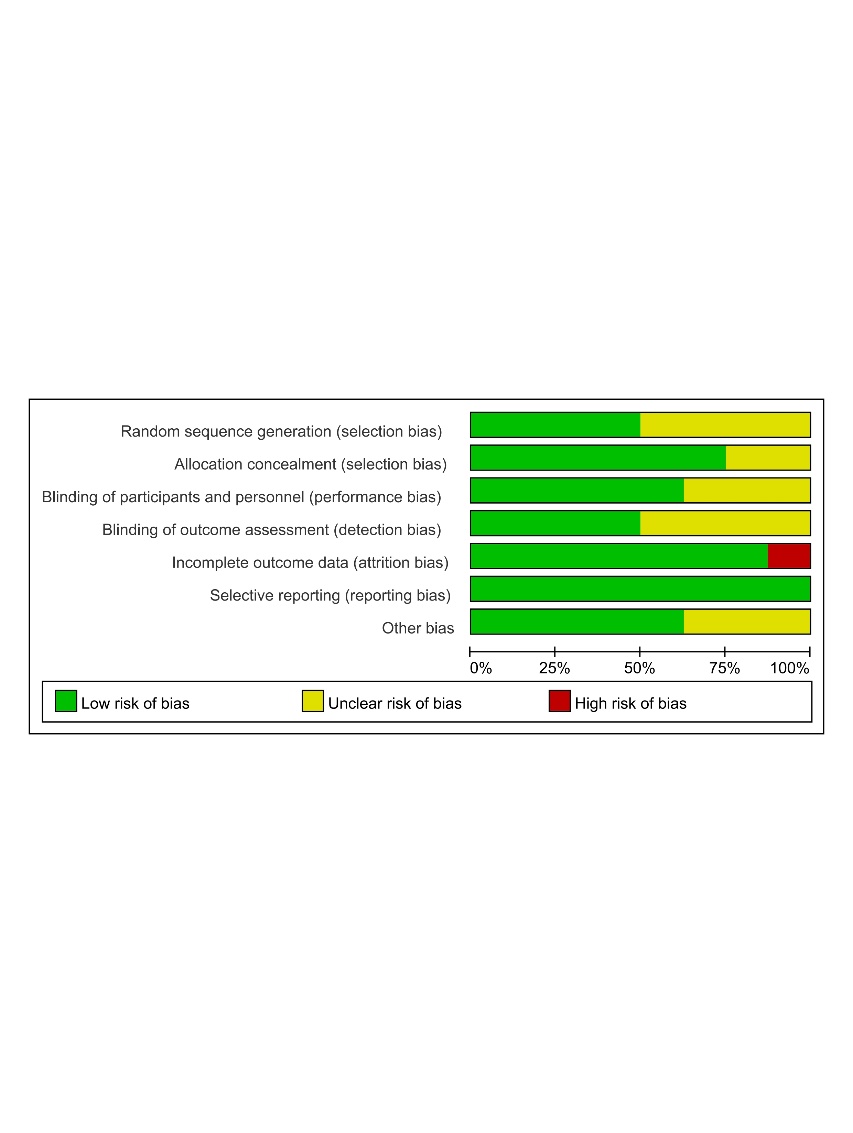

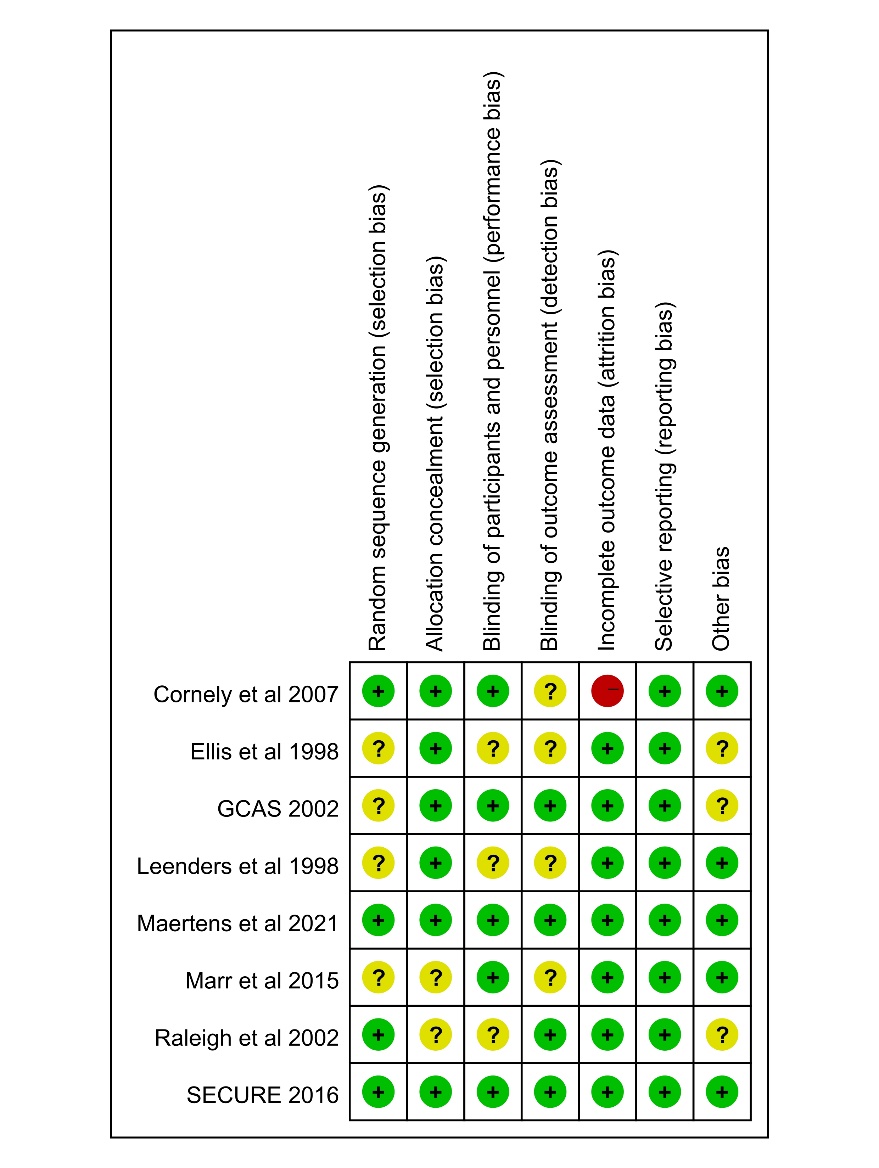


**Supplementary Figure 3 Summary of results from assessment of studies using the Cochrane risk of bias tool.**

**
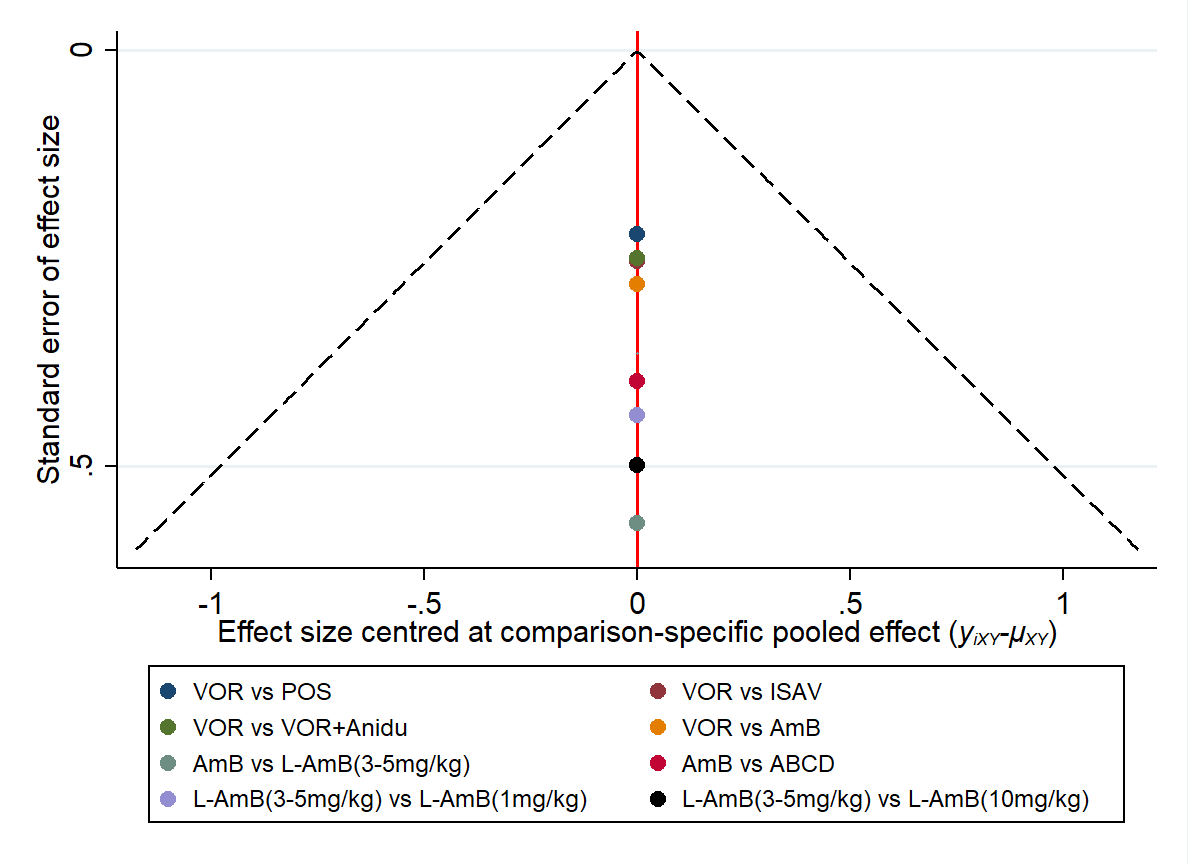
**

**Supplementary Figure 4. The funnel plot for detecting publication bias in the overall response.**

**
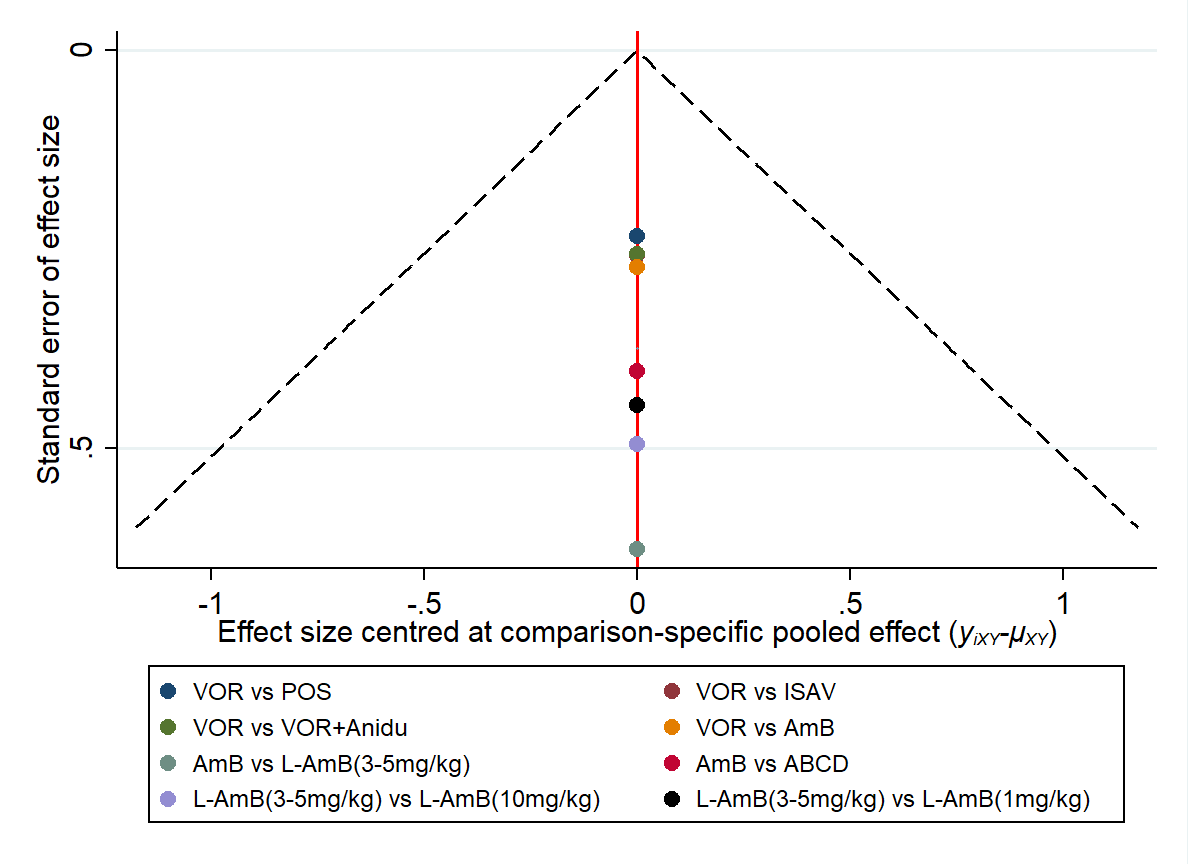
**

**Supplementary Figure 5. The funnel plot for detecting publication bias in the all-cause mortality (ACM).**
