# Supplementary material for: Comparison of antifungal drugs in the treatment of invasive pulmonary aspergillosis: a systematic review and network meta-analysis
Source: Front Microbiol. 2024 Dec 2;15:1504826. doi: 10.3389/fmicb.2024.1504826 (PMC11648418; doi:10.3389/fmicb.2024.1504826)
Supplement: Supplementary file 2 [file Table_2.docx]

**Author(s):** Jing Cheng, Hedong Han and Wenwen Kang

**Question:** different antifungal drugs compared to voriconazole or other drugs for invasive pulmonary aspergillosis

**Setting:**

**Bibliography:**

| **Certainty assessment** | | | | | | | **№ of patients** | | **Effect** | | **Certainty** | **Importance** |
| --- | --- | --- | --- | --- | --- | --- | --- | --- | --- | --- | --- | --- |
| **№ of studies** | **Study design** | **Risk of bias** | **Inconsistency** | **Indirectness** | **Imprecision** | **Other considerations** | **[intervention]** | **[comparison]** | **Relative (95% CI)** | **Absolute (95% CI)** |  |  |
| **Overall response (POS vs. VOR)** | | | | | | | | | | | | |
| 1 | randomised trials | not serious | not serious | not serious | not serious | none | 69/163 (42.3%) | 79/171 (46.2%) | **OR 0.85** (0.55 to 1.32) | **40 fewer per 1,000** (from 141 fewer to 69 more) | ⨁⨁⨁⨁ High | CRITICAL |
| **Overall response (ISAV vs. VOR)** | | | | | | | | | | | | |
| 1 | randomised trials | not serious | not serious | not serious | serious^a^ | none | 50/143 (35.0%) | 47/129 (36.4%) | **OR 0.94** (0.57 to 1.54) | **14 fewer per 1,000** (from 118 fewer to 105 more) | ⨁⨁⨁◯ Moderate | CRITICAL |
| **Overall response (VOR+Anidu vs. VOR)** | | | | | | | | | | | | |
| 1 | randomised trials | not serious | not serious | not serious | serious^b^ | none | 29/113 (25.7%) | 62/124 (50.0%) | **OR 0.64** (0.39 to 1.05) | **110 fewer per 1,000** (from 219 fewer to 12 more) | ⨁⨁⨁◯ Moderate | CRITICAL |
| **Overall response (dAmB vs. VOR)** | | | | | | | | | | | | |
| 1 | randomised trials | not serious | not serious | not serious | not serious | none | 29/113 (25.7%) | 62/124 (50.0%) | **OR 0.35** (0.20 to 0.60) | **241 fewer per 1,000** (from 333 fewer to 125 fewer) | ⨁⨁⨁⨁ High | CRITICAL |
| **Overall response (L-AmB 3-5mg/kg vs. dAmB)** | | | | | | | | | | | | |
| 1 | randomised trials | not serious | not serious | not serious | serious^c^ | none | 21/32 (65.6%) | 19/34 (55.9%) | **OR 1.59** (0.52 to 4.84) | **109 more per 1,000** (from 162 fewer to 301 more) | ⨁⨁⨁◯ Moderate | CRITICAL |
| **Overall response (L-AmB 3-5mg/kg vs. L-AmB 1mg/kg)** | | | | | | | | | | | | |
| 1 | randomised trials | not serious | not serious | not serious | serious^c^ | none | 22/46 (47.8%) | 22/41 (53.7%) | **OR 0.53** (0.22 to 1.25) | **156 fewer per 1,000** (from 334 fewer to 55 more) | ⨁⨁⨁◯ Moderate | CRITICAL |
| **Overall response (L-AmB 3-5mg/kg vs. L-AmB 10mg/kg)** | | | | | | | | | | | | |
| 1 | randomised trials | not serious | not serious | not serious | very serious^a,c^ | none | 18/45 (40.0%) | 16/38 (42.1%) | **OR 0.82** (0.31 to 2.17) | **47 fewer per 1,000** (from 237 fewer to 191 more) | ⨁⨁◯◯ Low | IMPORTANT |
| **Overall response (ABCD vs. dAmB)** | | | | | | | | | | | | |
| 1 | randomised trials | not serious | not serious | not serious | serious^b^ | none | 24/50 (48.0%) | 31/53 (58.5%) | **OR 0.66** (0.30 to 1.43) | **103 fewer per 1,000** (from 288 fewer to 83 more) | ⨁⨁⨁◯ Moderate | CRITICAL |
| **All-cause mortality (POS vs. VOR)** | | | | | | | | | | | | |
| 1 | randomised trials | not serious | not serious | not serious | not serious | none | 56/163 (34.4%) | 53/171 (31.0%) | **OR 1.17** (0.74 to 1.84) | **35 more per 1,000** (from 60 fewer to 143 more) | ⨁⨁⨁⨁ High | CRITICAL |
| **All-cause mortality (ISAV vs. VOR)** | | | | | | | | | | | | |
| 1 | randomised trials | not serious | not serious | not serious | serious^a^ | none | 43/143 (30.1%) | 48/129 (37.2%) | **OR 0.73** (0.44 to 1.20) | **70 fewer per 1,000** (from 165 fewer to 43 more) | ⨁⨁⨁◯ Moderate | CRITICAL |
| **All-cause mortality (VOR+Anidu vs. VOR)** | | | | | | | | | | | | |
| 1 | randomised trials | not serious | not serious | not serious | serious^b^ | none | 39/135 (28.9%) | 55/142 (38.7%) | **OR 0.64** (0.39 to 1.06) | **99 fewer per 1,000** (from 190 fewer to 14 more) | ⨁⨁⨁◯ Moderate | CRITICAL |
| **All-cause mortality (dAmB vs. VOR)** | | | | | | | | | | | | |
| 1 | randomised trials | not serious | not serious | not serious | not serious | none | 51/113 (45.1%) | 37/124 (29.8%) | **OR 0.52** (0.30 to 0.88) | **117 fewer per 1,000** (from 185 fewer to 26 fewer) | ⨁⨁⨁⨁ High | CRITICAL |
| **All-cause mortality (L-AmB 3-5mg/kg vs. dAmB)** | | | | | | | | | | | | |
| 1 | randomised trials | not serious | not serious | not serious | serious^c^ | none | 7/32 (21.9%) | 13/34 (38.2%) | **OR 0.39** (0.11 to 1.33) | **188 fewer per 1,000** (from 319 fewer to 69 more) | ⨁⨁⨁◯ Moderate | CRITICAL |
| **All-cause mortality (L-AmB 3-5mg/kg vs. L-AmB 1mg/kg)** | | | | | | | | | | | | |
| 1 | randomised trials | not serious | not serious | not serious | serious^c^ | none | 31/46 (67.4%) | 24/41 (58.5%) | **OR 1.46** (0.61 to 3.51) | **88 more per 1,000** (from 123 fewer to 247 more) | ⨁⨁⨁◯ Moderate | CRITICAL |
| **All-cause mortality (L-AmB 3-5mg/kg vs. L-AmB 10mg/kg)** | | | | | | | | | | | | |
| 1 | randomised trials | not serious | not serious | not serious | very serious^a,c^ | none | 19/45 (42.2%) | 19/38 (50.0%) | **OR 0.89** (0.34 to 2.36) | **29 fewer per 1,000** (from 246 fewer to 202 more) | ⨁⨁◯◯ Low | IMPORTANT |
| **All-cause mortality (ABCD vs. dAmB)** | | | | | | | | | | | | |
| 1 | randomised trials | not serious | not serious | not serious | serious^b^ | none | 18/50 (36.0%) | 24/53 (45.3%) | **OR 0.68** (0.31 to 1.50) | **93 fewer per 1,000** (from 249 fewer to 101 more) | ⨁⨁⨁◯ Moderate | CRITICAL |

**CI:** confidence interval; **OR:** odds ratio

#### Explanations

a. The study included a portion of patients infected with other non-Aspergillus molds

b. The study may include patients without pulmonary infection.

c. The research sample size was small.
